# Comparison of Breast Cancer Radiotherapy Techniques Regarding Secondary Cancer Risk and Normal Tissue Complication Probability – Modelling and Measurements Using a 3D-Printed Phantom

**DOI:** 10.3389/fonc.2022.892923

**Published:** 2022-07-27

**Authors:** Marc Vogel, Jonas Gade, Bernd Timm, Michaela Schürmann, Hendrik Auerbach, Frank Nüsken, Christian Rübe, Patrick Melchior, Yvonne Dzierma

**Affiliations:** ^1^ Department of Radiotherapy and Radiation Oncology, Saarland University Medical Centre, Homburg, Germany; ^2^ Siemens Healthcare GmbH, Technical Service, Erlangen, Germany

**Keywords:** radiation therapy, secondary cancer risk, normal tissue complication probability (NTCP), brachytherapy, breast cancer, 3D-printing

## Abstract

**Background:**

Radiotherapy after breast-conserving therapy is a standard postoperative treatment of breast cancer, which can be carried out with a variety of irradiation techniques. The treatment planning must take into consideration detrimental effects on the neighbouring organs at risk—the lung, the heart, and the contralateral breast, which can include both short- and long-term effects represented by the normal tissue complication probability and secondary cancer risk.

**Patients and Methods:**

In this planning study, we investigate intensity-modulated (IMRT) and three-dimensional conformal (3D-CRT) radiotherapy techniques including sequential or simultaneously integrated boosts as well as interstitial multicatheter brachytherapy boost techniques of 38 patients with breast-conserving surgery retrospectively. We furthermore develop a 3D-printed breast phantom add-on to allow for catheter placement and to measure the out-of-field dose using thermoluminescent dosimeters placed inside an anthropomorphic phantom. Finally, we estimate normal tissue complication probabilities using the Lyman–Kutcher–Burman model and secondary cancer risks using the linear non-threshold model (out-of-field) and the model by Schneider et al. (in-field).

**Results:**

The results depend on the combination of primary whole-breast irradiation and boost technique. The normal tissue complication probabilities for various endpoints are of the following order: 1%–2% (symptomatic pneumonitis, ipsilateral lung), 2%–3% (symptomatic pneumonitis, whole lung), and 1%–2% (radiation pneumonitis grade ≥ 2, whole lung). The additional relative risk of ischemic heart disease ranges from +25% to +35%. In-field secondary cancer risk of the ipsilateral lung in left-sided treatment is around 50 per 10,000 person-years for 20 years after exposure at age 55. Out-of-field estimation of secondary cancer risk results in approximately 5 per 10,000 person-years each for the contralateral lung and breast.

**Conclusions:**

In general, 3D-CRT shows the best risk reduction in contrast to IMRT. Regarding the boost concepts, brachytherapy is the most effective method in order to minimise normal tissue complication probability and secondary cancer risk compared to teletherapy boost concepts. Hence, the 3D-CRT technique in combination with an interstitial multicatheter brachytherapy boost is most suitable in terms of risk avoidance for treating breast cancer with techniques including boost concepts.

## 1 Introduction

Regarding the female sex, breast carcinoma was both the most frequent entity of all new cancer incidences and the most frequent cause of mortality of all cancer deaths in Europe in 2018 (1, 2). Due to this importance for society as a whole, screening programmes, targeted diagnostics, and a wide variety of therapy regimes are standard today and are subject to constant testing and further refinement. Adjuvant radiotherapy in the context of breast-conserving therapy (BCT) has been shown to be an indispensable component of the therapy regime. A meta-analysis of the Early Breast Cancer Trialists’ Collaborative Group in 2011 found a significant reduction in the risk of recurrence within 10 years for adjuvant radiotherapy vs. no adjuvant radiotherapy (3). The German Society for Radiation Oncology (DEGRO) also clearly advocates adjuvant radiation in its guideline recommendation for the treatment of breast carcinoma, and at the same time, it emphasises the importance of additional dose saturation (boost) to the tumour bed in order to further reduce the risk of local recurrence (4).

For adjuvant breast irradiation (whole-breast irradiation, WBI), the procedures three-dimensional conformal radiotherapy (3D-CRT), intensity-modulated radiotherapy (IMRT), and volume-modulated arc therapy (VMAT) are currently mentioned in the S3 guidelines of the German Cancer Society, German Cancer Aid, and Association of the Scientific Medical Societies in Germany (AWMF) (5). 3D-CRT was the treatment standard until the 2010s but is increasingly being replaced by IMRT or VMAT, especially due to the higher computing power of the available computer hardware and improved planning software (6, 7). With regard to an additional dose saturation of the tumour bed, external irradiation procedures are available on the one hand, for example additive percutaneous irradiation sessions (sequential boost) following the total breast irradiation (WBI) or by means of a simultaneous integrated boost (SiB), in which the boost saturation is included in the percutaneous WBI (8–11). On the other hand, boost treatment can be applied by means of brachytherapy (12), e.g., using the afterloading technique with interstitial catheters, so that the additional desired dose can be deposited in the tumour bed over the course of several treatment sessions. The prognostic benefit of a boost is considered certain and advocated for patients especially with an elevated risk for local relapse (age < 40–50 years) and for older patients with an elevated risk for local recurrence (G3, HER2+, triple negative, > T1). Bartelink et al. (13) were able to show that an additional dose escalation of the tumour bed with 16 Gy significantly reduces the 5-year local recurrence rate from 7.3% to 4.3% compared to a comparison group without boost irradiation. Kindts et al. (14) reached the same conclusion in a systematic review in 2017, which found a hazard ratio of 0.64 for local 5-year tumour control with boost irradiation. With regard to the superiority of a boost technique combined with percutaneous WBI for reducing the risk of local recurrence (percutaneous boost versus brachytherapy boost), technical subgroup analyses from the EORTC trial 22881/10882 by Portmans et al. (15) and retrospective studies from Bartelink and Hammer et al. (13, 16, 17) assumed a potential clinical advantage concerning local control and better cosmetic results in favour of integrated brachytherapy boost concepts.

While high-dose delivery to the planning target volume (PTV) are aimed for and desired, in return the requirement is to avoid or keep as low as possible the dose deposition in surrounding organs at risk (OAR) or normal tissue in order to avoid damage by ionising radiation. Even if the benefit of the adjuvant radiation regime in terms of tumour control, recurrence risk, and overall survival is significant, radiotherapeutic side effects in normal tissue must be taken into account as critical factors in treatment planning. This applies in particular to the OAR skin, heart, lungs, and the contralateral breast. Common or frequently described clinical findings are above all cosmetic damage, radiation dermatitis, and breast fibrosis as well as cardiac ischemic damage and radiation pneumonitis (18, 19). It must also be borne in mind that ionising radiation may induce second primary cancer (20–22), which is strongly dependent on the combination of the treatment concepts.

In this paper, we compare several standard breast treatment techniques with respect to dose, normal tissue complication probability, and secondary cancer risk. Given the fact that the different boost irradiation techniques have hitherto not been observed to differ in clinical benefit, we aim to address the question whether they do regarding treatment-related sequelae. To achieve this, both 3D-CRT and IMRT techniques with sequential and simultaneously integrated teletherapy boost and afterloading multicatheter brachytherapy boost are considered for a collective of patients treated for left-sided breast cancer at our department. A dosimetric comparison includes the summation dose from the WBI and boost plans (corrected for different brachytherapy fractionation), which is used as input for the normal tissue complication probabilities (*NTCP*) and in-field (100% to 80% isodose area) to penumbra (80% to 20% isodose area, defined in ref. 24) secondary cancer risk models to account for the high-dose areas. In the low-dose regime outside the treatment beams (below 5% isodose area), the dose computations from the treatment planning systems are generally unreliable. Hence, for the out-of-field regions far from the primary beams, dose measurements are performed in an anthropomorphic phantom with realistic breast attachments created by 3D printing for afterloading catheter insertion. The measured average organ doses are then translated into secondary cancer risk using the linear non-threshold model. To our knowledge, this is the first study to include this comprehensive modelling and measuring approach for assessing the differences between these widespread breast treatment techniques in a realistic setting. Similar studies comparing late side effects with various techniques for different entities can be found in literature, e.g., the prostate (23).

## 2 Materials and Methods

### 2.1 Study Design

Four different treatment scenarios are considered here:

*scenario 1:* 3D-CRT WBI treatment (25 × 2 Gy to a total dose of 50 Gy) planned using tangential beams with an additional sequential boost of 5 × 2 Gy using three beams.
*scenario 2:* 3D-CRT WBI treatment as above (25 × 2 Gy), followed by an interstitial multicatheter brachytherapy boost of 2 × 6 Gy.
*scenario 3:* IMRT treatment (fanned tangents, step-and-shoot technique) of 25 × 2-Gy fractions applied with up to eight beams, planned using direct machine parameter optimisation (DMPO), followed by an interstitial multicatheter brachytherapy boost of 2 × 6 Gy.
*scenario 4:* IMRT treatment with a SiB concept fractionated as 28 × 1.8 Gy to the whole breast and 28 × 2.14 Gy to the tumour bed.

**Figure 1:**
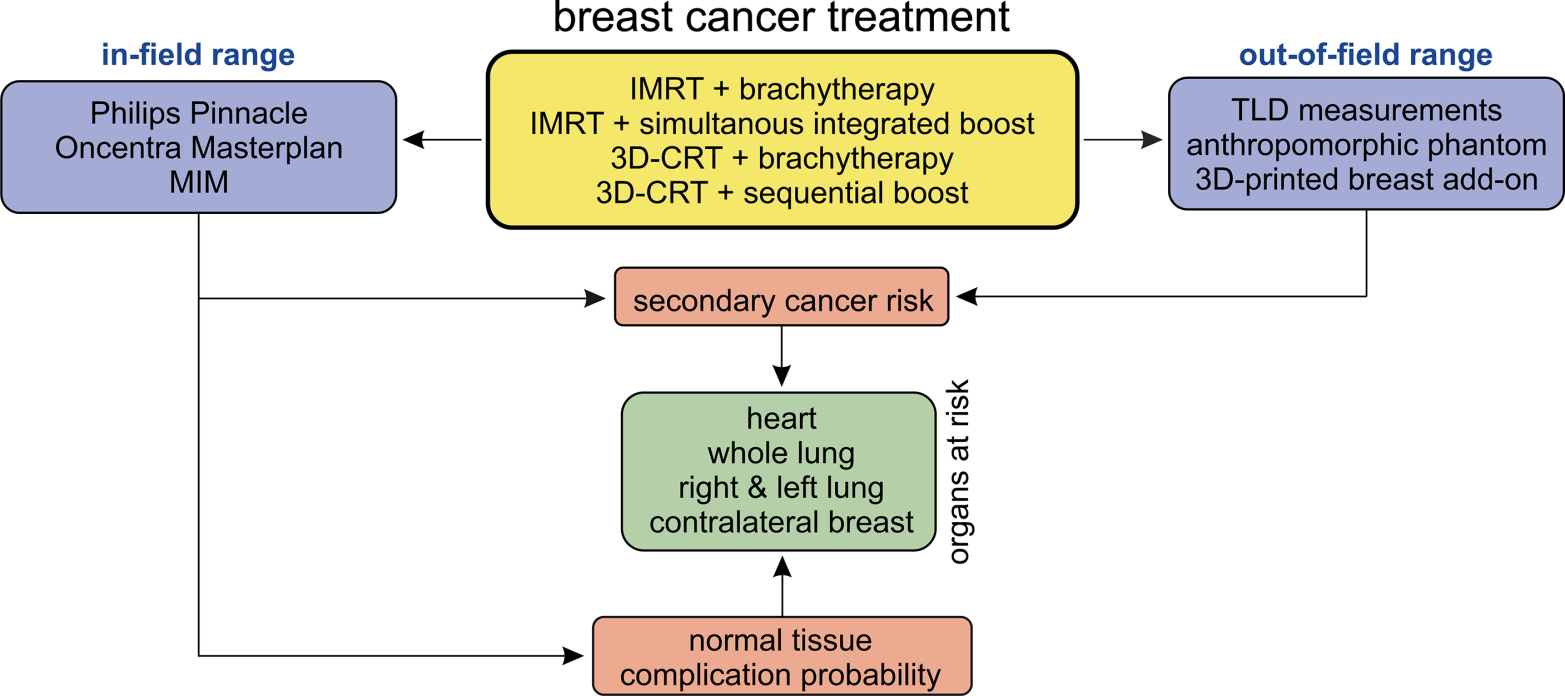
Schematic of the study design.

The study design is shown schematically in **Figure 1**. In the in-field region, the investigation is performed retrospectively on the basis of the patients’ CT data sets and calculated summation treatment plans. Since the treatment planning system (TPS, discussed in Section 2.3) is not intended to provide accuracy in the out-of-field region (24–27), we here perform thermoluminescent dosimeter (TLD) measurements in an anthropomorphic phantom. The respective plans are irradiated on the phantom, and dose measurements are carried out using the TLDs. After the TPS calculations, the *NTCP* for various endpoints, as well as the secondary cancer risk using the TLD measurements (out-of-field) and TPS calculations (in-field and penumbra), are determined. We investigate the exposure on all relevant OAR for breast cancer treatment—the heart, the contralateral breast, the ipsilateral and contralateral lung, and the whole lung. Based on this, the *NTCP* for various endpoints and the secondary cancer risk are determined.

### 2.2 Patient Cohort

Treatment plans for 38 patients with left-sided breast cancer treated at our institution after breast-conserving surgery between January 2011 and December 2019 were retrospectively included in our study. The selection of patients was based on the fact that in 2011, 3D-CRT with percutaneous or afterloading interstitial multicatheter brachytherapy boost was the standard treatment regime, and consecutive patients out of this collective were chosen. By 2019, most patients were treated either using IMRT + SiB or using IMRT + brachytherapy, so the last consecutive patients out of these ensembles were selected. It was tried to exclude patients from the transitional phase in which the new techniques were being established and hence still subjected to adjustments and improvements. January 2011 was taken as the starting time for study inclusion since this corresponds to the installation of a new set of three linear accelerators, afterloading unit, and CT scanner, all of which remained functional and in clinical use until the end of 2019, so that the same machines and treatment planning systems were used for all patients in this study. A target of 10 patients per scenario was set. For the cohort “3D-CRT + brachytherapy boost”, however, this resulted in only eight cases, as 3D-CRT was replaced by IMRT as standard in our clinic during the observation period.

Across the cohort of 38 patients, tumour gradings ranged from G1 to G3 and the tumour stages were pT1b/pT1c/pT2 pN0 cM0 L0 R0. In six cases, the diagnosis was pN1, in seven cases L1, and once an M1 diagnosis was made. The adjuvant or neoadjuvant systemic therapy was selected based on the usual guidelines regarding tumour stage and grade, patient age, hormone receptor expression (ER/PR) and menopausal status for endocrine therapy, and HER2-expression for targeted therapy (trastuzumab/pertuzumab). Chemotherapy was mainly applied using the EC regime (epirubicin, cyclophosphamide), often in combination with either paclitaxel/docetaxel, carboplatin, or 5-FU. Due to the relatively small collectives, no significant difference in systemic therapy regimes can be proven amongst the four scenarios. Overall, only two patients did not receive any systemic therapy at all. Eighteen patients (47%) received chemotherapy, four of whom in a neoadjuvant setting. Endocrine therapy was given in 28 cases (74%), targeted therapy in three patients (8%). The age of the patients varies between 35 and 76 years (median value: 52 years, mean value: 55 years). The cohort-related median/mean values are as follows: “IMRT + brachytherapy” (49/48 years), “IMRT + SiB” (59/59 years), “3D-CRT + brachytherapy” (46/46 years), and “3D-CRT + sequential boost” (64/61 years). A comparison of the two scenarios with brachytherapy boost shows no significant statistical difference in age (t-test: p = 0.420). The same applies for the scenarios using teletherapy boosts (t-test: p = 0.610). All other pairwise t-tests result in p ≤ 0.05, i.e., patients receiving brachytherapy were significantly younger. Furthermore, comparing the breast and PTV volumes of the various cohorts show no significant statistical difference.

### 2.3 Treatment Planning and Treatment Machines

All percutaneous treatment plannings involved an in-house-acquired dedicated planning CT (Philips Brilliance Big Bore, 120 kV, Philips Healthcare, Amsterdam, Netherlands) dataset with the patients positioned supine with their arms raised above the head. The data were imported into the Philips Pinnacle treatment planning system (V. 9.0-9.8, 14.0, 16.0, and 16.2, Philips Medical Systems, Fitchburg, Wisconsin, USA), and treatment plans were created depending on the planning scenario (see below) for the three linacs available at our department. Dose calculation was performed using the collapsed cone convolution (CC) algorithm on a 2 × 2 × 2 mm³ dose grid. The percutaneous radiotherapy of the patients was administered using a Siemens Oncor and two Siemens Artiste linear accelerators (Siemens Healthcare, Erlangen, Germany) with identical 160 multi-leaf collimators. The beam energies for treatment were 6 and 18 MV, with beam matching amongst all machines for 6 MV and between one Artiste and the Oncor for 18 MV (28).

The 3D-CRT treatment plans for WBI used tangential beams with 6- and 18-MV mixed energies with dynamic wedges, with beam and couch angles adjusted to eliminate beam divergence in the lung. The 3D-CRT boost plan used three 6-MV beams in a field-in-field technique. IMRT plans (both for WBI and for SiB) involved up to eight beams with 6 MV and the step-and-shoot technique with direct machine parameter optimisation (DMPO) based on our in-house template of objectives, which is given in **Table 1**. The beams were distributed in a fan-like pattern depending on the patient anatomy, excluding beam angles through the back of the patient or the contralateral breast.

**Table 1:** Organs at risk TPS dose statistics versus planning objectives.

		*scenario 1*	*scenario 2*	*scenario 3*	*scenario 4*
organ at risk	objectives	IMRT + brachytherapy	IMRT + SiB	3D-CRT + brachytherapy	3D-CRT + sequential boost
**heart**	*D*_mean_ < 3 Gy	4.5 ± 1.3 (3.3 – 7.9)	4.4 ± 1.0 (2.8 – 6.1)	3.3 ± 1.6 (1.5 – 6.0)	4.8 ± 2.1 (2.4 – 7.6)
					
**left lung (ipsilateral)**	*D*_mean_ < 12 Gy	9.9 ± 2.3 (7.5 – 14.9)	10.8 ± 1.8 (7.5 – 13.7)	7.9 ± 1.1 (6.6 – 9.9)	8.7 ± 2.2 (5.0 – 13.5)
		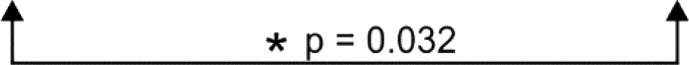			
			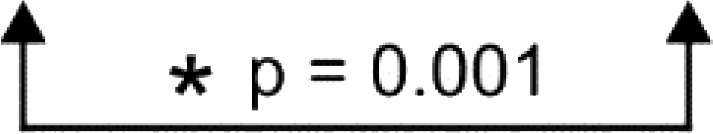		
			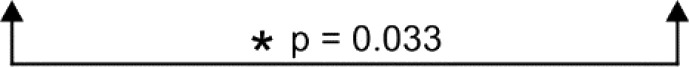		

**whole lung**	*D*_mean_ < 10 Gy	5.3 ± 1.6 (4.0 – 9.5)	5.9 ± 1.3 (4.3 – 9.1)	3.9 ± 0.8 (3.0 – 5.3)	4.2 ± 1.1 (2.4 – 6.6)
		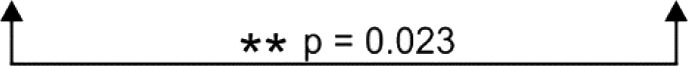			
		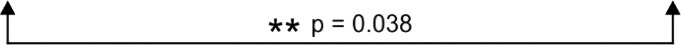			
			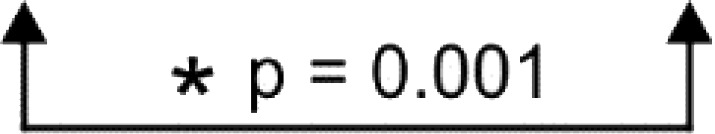		
			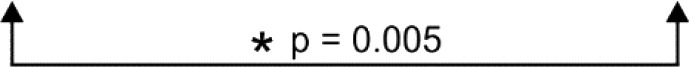		
	*V*_20 Gy_ < 10%	7.4 ± 1.6 (5.3 – 10.2)	8.6 ± 1.9 (4.9 – 11.9)	6.1 ± 1.3 (4.7 – 8.3)	6.3 ± 2.0 (3.1 – 10.2)
			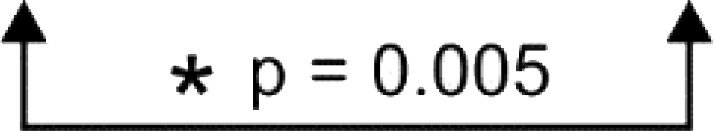		
			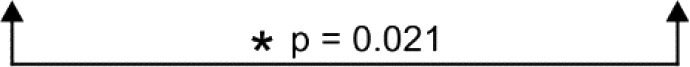		

**right breast (contralateral)**	*D*_1%_ [Gy] minimised	9.6 ± 8.3 (3.0 – 24.7)	8.5 ± 12.2 (2.7 – 42.7)	3.4 ± 2.0 (1.2 – 8.0)	3.5 ± 1.3 (1.6 – 6.1)
		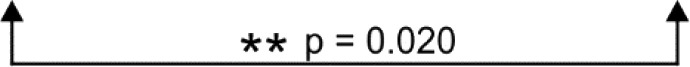			
		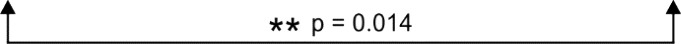			
			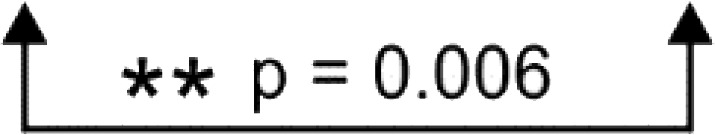		
			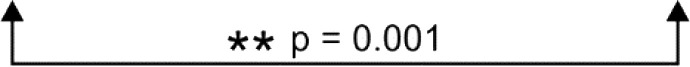		
	*D*_mean_ [Gy] minimised	2.3 ± 2.2 (1.1 – 8.2)	2.5 ± 2.1 (1.2 – 8.4)	0.7 ± 0.7 (0.1 – 2.2)	0.8 ± 0.6 (0.3 – 2.1)
		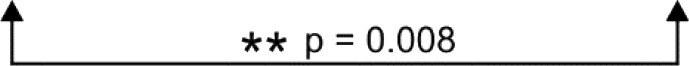			
		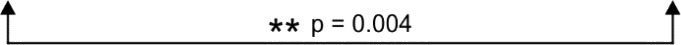			
			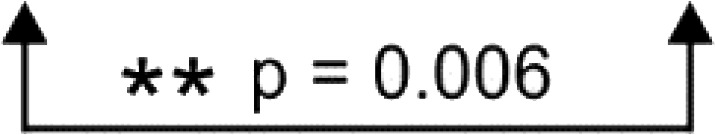		
			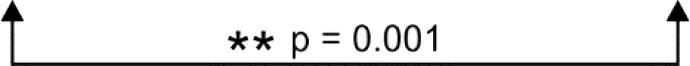		

*marks a pairwise t-test with p ≤ 0.05 including a Welch correction for different variances.**marks a pairwise Mann-Whitney U test with p ≤ 0.05.

For the interstitial multicatheter brachytherapy boost, up to 16 catheters were implanted in the patient breast after the end of the percutaneous WBI series, based on the pre- and postoperative imaging information and positioning of titan clips on the localisation of the tumour bed. A planning CT was acquired using the same Philips Big Bore CT as for teletherapy planning, and the data were transferred to the Oncentra Masterplan TPS (version 4.6.0, Nucletron B.V., Veenendaal, Utrecht, Netherlands) for planning. The afterloader to apply the additional brachytherapy boost was a Flexitron (Elekta, Hamburg, Germany) using radioactive Iridium-192.

### 2.4 Plan Summation and Dosimetric Analysis

To compare all these scenarios with different fractionation schemes, the isoeffective total dose or biological effective dose (*BED*) must be considered (29):


[1] $$BED=n\cdot d_{\text{T}}\cdot \left(1+\frac{d_{\text{T}}}{\alpha /\beta }\right),$$

where *α* and *β* are the coefficients used in the linear–quadratic model, *d*_T_ is the single fraction dose, and *n* is the number of fractions. Since high-dose-rate (HDR) brachytherapy is considered here, the influence of protracted irradiation can be neglected as shown in Equation [1] (30). For late-responding tissue, we use the approximation *α*/*β* = 3 (also used in the full model to calculate the secondary cancer risk; see Section 2.6.2).

The analysis software MIM (version 6.8.7, MIM Software Inc., Cleveland, OH, USA) was used to merge the planning CT images of teletherapy and brachytherapy treatment and thereby create summation plans. Deformable image registration from the percutaneous and brachytherapy planning CT datasets was performed by manually adjusting the automatically registered images so that the position of the left breast and the adjacent lungs showed best agreement. The registration result was independently verified by a senior radiation oncologist. The in-field dose distribution from Pinnacle and MIM was used to assess the dosimetric parameters of the plans, i.e., the mean heart dose, the mean dose to the ipsilateral and whole lung, *V*_20 Gy_ of the lung, *D*_1%_ of the contralateral breast (as an estimate of the maximum dose), and the mean dose to the contralateral breast. The dose distributions were then used as an input to model the *NTCP* and secondary cancer risk as explained in Section 2.6.

### 2.5 Measurements of Out-of-Field Doses

In the out-of-field regions, commercially available TPSs commonly underestimate the real dose, with calculation accuracy decreasing with distance from the field edge (24–27). This is due to the dose calculation algorithm (collapsed cone convolution superposition in our case) which uses in-field kernel approximations to determine the dose distribution and can be circumvented by advanced algorithms such as grid-based Boltzmann solvers or Monte Carlo calculations. Furthermore, the CCC algorithm does not realistically account for head leakage, collimator scatter, and patient scatter. Consequently, TLD-100H disks (Thermo Fisher Scientific, Waltham, MA, USA) were used to measure the dose out-of-field, using a Harshaw TLD 5500 reader (Thermo Fisher Scientific). The calibration and measurement settings for the TLDs have been described elsewhere (31). In short, the vendor-recommended time–temperature protocol was used, which presumes 5 s of preheating at 145°C, followed by acquisition at 10°C/s up to a maximum temperature of 260°C for 23 1/3 s, and finally annealing at 260°C for 20 s. In all four scenarios, the out-of-field measurements were carried out inside an anthropomorphic phantom with breast attachments representing either a plausible large breast or small breast size (CIRS Atom Dosimetry Verification Phantom Model 701). Breast attachments with 350 and 1200 cc were selected, so that for each treatment scenario, the patient cohort was searched for two patients best matching the phantom anatomy and breast sizes—these patients were used for phantom measurements.

**Figure 2:**
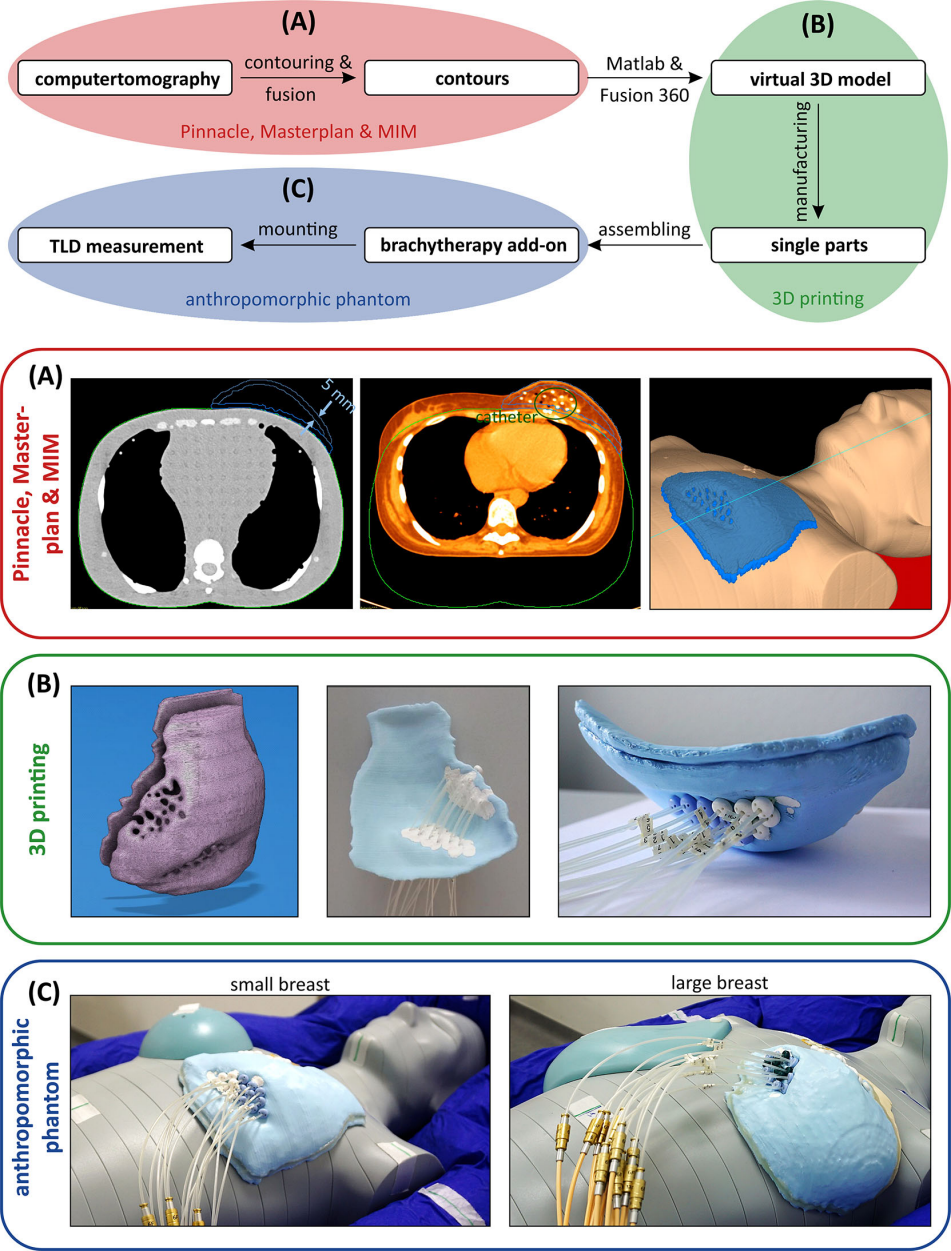
Schematic process to fabricate a brachytherapy breast add-on for the anthropomorphic phantom to measure the dose in the out-of-field range using thermoluminescent dosimeters. **(A)** Fusioning the brachytherapy planning and the anthropomorphic phantom CT to create contours as a starting point for the manufacturing. **(B)** From a virtual 3D model to a manufactured breast model. **(C)** Mounting the small (left) and large (right) breast models to the anthropomorphic phantom and the afterloader.

**Figure 3:**
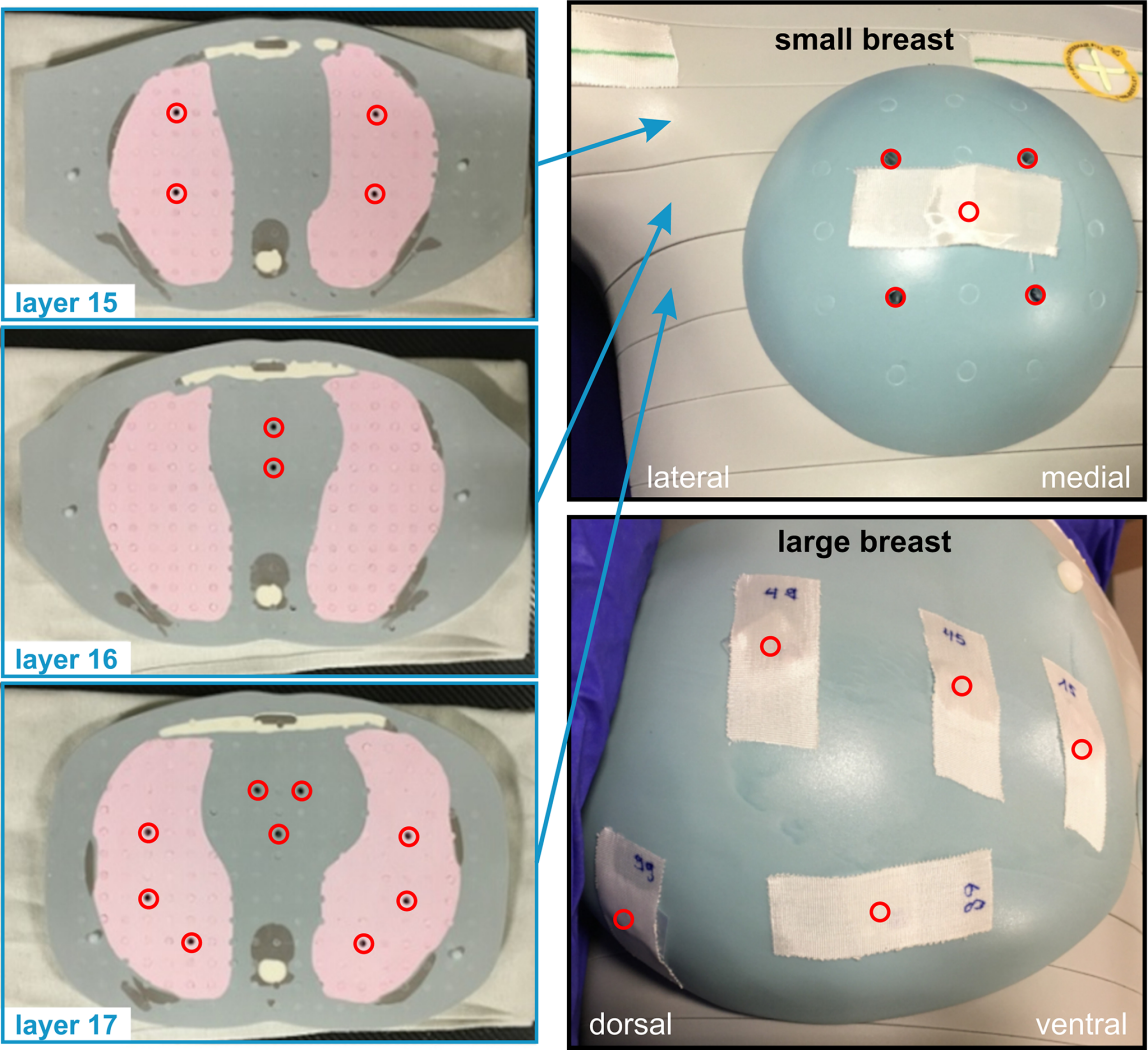
TLD placement inside and on the surface of the anthropomorphic phantom. In the superior–inferior direction, different phantom layers with numbers 15 to 17 are shown on the left side of the image. The TLD positions are depicted as red circles. On the right side, the placement regarding the small and large breast add-ons is shown.

Using the MIM fusions, the teletherapy treatment plans could be mapped onto the phantom straight forward. For the experimentally more complex brachytherapy sub-cohorts, we developed 3D-printed phantom breast add-ons, since catheter insertion would not have been possible in the CIRS phantom breast attachments and also since the catheters result in deformation of the patient breast, which would not have been realistically reproduced by the phantom. Two representative breast models were reconstructed using CT data sets (one small and one large left breast) in order to allow for multicatheter placement. The manufacturing process is depicted in **Figure 2**. We performed a DICOM export of the CT fusion from MIM towards Pinnacle, where regions of interest (ROIs) were contoured (see blue contours in **Figure 2A**, left and centre). To create the outer shape of the breast add-on, we used the skin contour of the patient brachytherapy CT and subtracted the co-registered body contour of the phantom CT for each CT layer. Afterwards, inner rings of 5 mm in diameter were created to realise a shell with 5-mm thickness, which was divided manually into front and backside part. All catheters were also contoured and subtracted from the ROI of the breast add-on in order to locate the catheter placement holes during the next step of the fabrication process. The final add-on contour for the small breast is shown in **Figure 2A** on the right side as a three-dimensional reconstruction. Now, the created structures were exported from Pinnacle to a DICOM node and manually imported into the Matlab software (version R2019b, MathWorks, Natick, MA, USA) to create a stereolithography point cloud. Afterwards, the software Fusion 360 (version 2.0.10148, Autodesk, San Rafael, CA, USA) was utilised to connect the points and to create a virtual computer-aided design (CAD) model (see **Figure 2B**, left). In the next fabrication step, we used the CAD to manufacture the single parts of the add-on using a commercially available 3D printer Prusa i3 MK3S+ (Prusa Research, Prague, Czech Republic). The printing layer thickness is 200 µm, the printing speed is 40 mm/s, and the filling factor is equal to 100%. We used 3DJAKE ecoPLA (niceshops GmbH, Paldau, Germany) as 3D-printing filament (mass density 1.24 g/cm³). Next, the catheters were placed in the predefined holes (see **Figure 2B**, centre), the front part was filled with white petroleum jelly (CAELO-PRIMA Vaseline, Caesar & Loretz GmbH, Hilden, Germany), and the printed backside part was used to assemble and seal the breast add-on (see **Figure 2B**, right). We use this filling material to model the breast in a most realistic way, since the jelly has a mass density of 0.9 g/cm³, which is very close to the density mean value of the representative breasts (0.89 ± 0.09 g/cm³). Both shells were fixated together using hot glue. In the third creation step of the brachytherapy add-on, we finally mounted the assembled parts to the anthropomorphic phantom and connected each catheter to the afterloader for irradiation. The overall result is shown in **Figure 2C** for the small (compare right inset of **Figure 2A**) and the large brachytherapy breast phantom. For all measurements (and prior to mounting the breast attachments), the TLDs were placed at representative places inside the phantom to determine the mean dose of each OAR. For this purpose, we used 20 TLDs—five per organ at risk (see **Figure 3**). Additionally, we included into the measurement three further TLDs which were placed outside the treatment room in order to measure the background radiation and subtract this from the measurement TLDs irradiated in the phantom. The localisation of the TLDs is also shown in **Figure 3**. The phantom was positioned inside a vacuum cushion with laser markings for better reproducibility of the measurements.

In total, we performed eight TLD measurements (one plan for a large and one for a small breast size for each of the four treatment scenarios) to determine the mean organ doses of the contralateral breast, the lung, and the heart. The data obtained are used for secondary cancer risk estimation only.

### 2.6 *NTCP* and Secondary Cancer Risk Modelling

Modelling secondary-cancer risk for low radiation doses usually relies on the linear non-threshold (LNT) assumption. In the high-dose regime, additional effects such as cell killing must be taken into account. A well-established secondary cancer risk model in the radiotherapeutic dose regime is the full (mechanistic) model by Schneider et al. (32–34). Schneider’s approach is based on the linear–quadratic model of dose response which is fitted to combined empirical data including patients treated for Hodgkin’s lymphoma and atomic bomb survivors to best adjust the available data in both the high- and low-dose ranges. In the low-dose limit, Schneider’s model is equivalent to the LNT assumption. Both models are described in more detail in the following subsections. For secondary cancer risk estimation of the contralateral breast and lung, we use the TLD data. Here, the expected isodoses are below 20% and therefore out-of-field. On the other hand, the ventral part of the ipsilateral lung is exposed to isodoses up to 80% since the PTV is directly adjacent. Hence, the secondary cancer risk mainly originates from this high-dose exposure and the risk estimation relies on TPS data only.

Modelling of the normal tissue complication probability (*NTCP*) can be achieved via, e.g., the Lyman–Kutcher–Burman model (35–37). Here, a DVH is needed to calculate the *NTCP*, which results from the high-dose burden onto the organ. Thus, we only use the TPS data for risk estimation. From a clinical point of view, the paper of Emami et al. defined the first guidelines on *NTCP* in 1991 (38). Due to the technical improvement in the field of radiotherapy since then, the Quantitative Analysis of Normal Tissue Effects in the Clinic (QUANTEC) review summarises the currently available guidelines (39–41). Additionally, there are specific models for ischemic heart disease, which rely on a linear approach (42).

#### 2.6.1 Linear Non-threshold Model—Out-of-field Secondary Cancer Risk

For low doses, the linear non-threshold model is generally accepted (43). We apply the parameterisation by Schneider for consistency with the high-dose regime (Section 2.6.2), where we also use the parameters proposed by the same authors (32–34).


[2] $$EAR\left(D,age_{x},age_{a}\right)=\delta \cdot D\cdot \mu \left(age_{\text{x}},age_{\text{a}}\right),$$

where *μ* is an exponential function depending on the age of exposure (*age*_x_) and the attained age (*age*_a_) according to


[3] $$\mu \;\left(age_{\text{x}},age_{\text{a}}\right)=\exp\left(\gamma_{\text{e}}\cdot \left(age_{\text{x}}-30\right)+\gamma_{\text{a}}\cdot \ln\left(\frac{age_{\text{a}}}{70}\right)\right).$$

γ_e_ and *γ*_a_ are organ-specific fit parameters. Furthermore, *δ* is the initial slope. In the out-of-field region, *D* is taken to be the mean organ dose. The average organ doses are determined from our TLD measurements in the contralateral breast and lungs (both ipsi- and contralateral).

#### 2.6.2 Schneider’s Full Model—in-field Secondary Cancer Risk

For the high-dose region inside the field and around the field edge, let us now discuss Schneider’s full model (32). Equation [2] is modified to account for the in-field region and cell regeneration between two fractions as follows:


[4] $$EAR\left(D,age_{x},age_{a}\right)=\delta \cdot RED\left(D\right)\cdot \mu \left(age_{\text{x}},age_{\text{a}}\right).$$

*RED* is the risk equivalent dose, which models the rate of cell regeneration:


[5] $$RED\left(D\right)=\frac{\text{exp}\left(-\alpha^{\prime }\cdot D\right)}{\alpha^{\prime }\cdot R}\cdot \left[1-2R+R^{2}\cdot \text{exp}\left(-\alpha^{\prime }\cdot D\right)-{\left(1-R\right)}^{2}\cdot \text{exp}\left(-\alpha^{\prime }\cdot D\cdot \frac{R}{1-R}\;\right)\right].$$

*α'* is also a function of the total dose *D* . Moreover, the term *α'* · *D* represents the linear–quadratic model of dose response including fractionation schemes, where


[6] $$\alpha^{\prime }\left(D\right)=\alpha +\beta \cdot \frac{d_{\text{T}}}{D_{\text{T}}}\cdot D.$$

**Figure 4:**
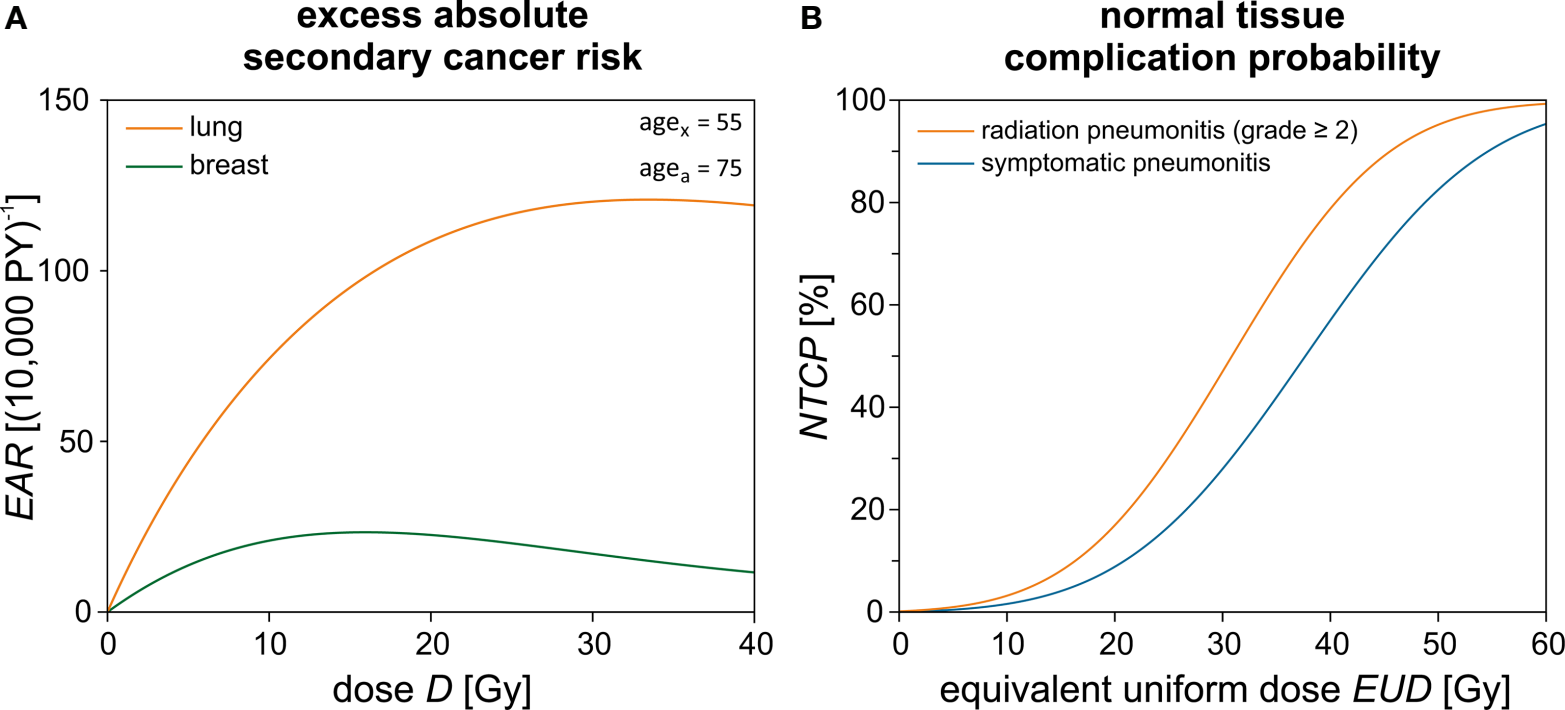
Theoretical risk estimation using empirical models. **(A)** The excess absolute secondary cancer risk (*EAR*) is calculated for the lung and the breast dependent on the dose *D* using Schneider’s model. The difference between the age exposed (*age*_x_) and the attained age (*age*_a_) is 20 years. **(B)** Normal tissue complication probability (*NTCP*) calculation for the lung and its respective endpoints dependent on the equivalent uniform dose (*EUD*).

The repopulation or repair capacity *R* of the tissue between two radiation fractions can take values between “0” (no regeneration) and “1” (complete regeneration). Both *α'* and *R* are organ- or tissue-specific parameters fit to the observations from the atomic bomb survivors data (low-dose exposure) and Hodgkin’s lymphoma patients (high-dose radiotherapy). In **Table 2**, these parameters are listed exemplarily for the lung and the breast. Equation [4] is plotted in **Figure 4A** for *age*_x_ = 55 years (mean value of the overall cohort) and *age*_a_ = 75 years. The unit of *EAR* is per person years (PY**^-1^**). The specific *EAR* of the OAR is finally computed in Matlab using the TLD measurements in the out-of-field regions and the in-field TPS dose distributions using an in-house Matlab script as described in a previous publication (44).

Please note that evidently only a small part of the ipsilateral lung and possibly a small medial portion of the contralateral breast may be included in the treatment field and therefore inside the “high-dose region”. Only for this region is the Schneider model applied to assess the secondary cancer risk according to the isodose distribution from the TPS. For the major part of the volume of the lungs and breast, only out-of-field leakage and scattered radiation will contribute to the dose. In this low-dose region, the TLD measurements are used to assess the mean organ dose and calculate the secondary cancer risk using the LNT models for the organ dose. The two approaches are chosen with the intention to give a lower and upper estimate of the plausible secondary cancer risk in these organs located close to steep-dose gradients. An example dose–response relationship for Schneider’s model is shown in **Figure 4**.

#### 2.6.3 Lyman–Kutcher–Burman Model for Normal Tissue Complication Probabilities

There are various mathematical models for estimating and modelling the risks of biological side effects. A common model, which is used in this work due to OAR parameter availability in literature, is the *NTCP* approach according to Lyman–Kutcher-Burman (35–37), which is expressed as follows:


[7] $$NTCP\left(EUD\right)=\;\frac{1}{\sqrt{2\pi }}\int_{-\infty }^{u\left(EUD\right)}exp\;\left(\frac{-t^{2}}{2}\right)\;dt.$$

The integrand describes a probability density function for the standard Gaussian distribution. The upper integral limit *u* is a function of the equivalent uniform dose *EUD* (45):


[8] $$u\left(EUD\right)=\frac{EUD-D_{50\%}}{m\;\cdot \;D_{50\%}},$$

where


[9] EUD=(∑ivi·Di1n)n.

The *EUD* describes a uniform dose which leads to the same complication risk as caused by the given non-uniform dose distribution. *m* represents the slope of the *NTCP* curve, and *D*_50%_ is the uniform dose, which applied to the entire organ volume would result in 50% risk of complication. *v_i_* is the *i*-th relative sub-volume receiving the dose *D_i_*. Both values are determined by the dose–volume histogram (DVH) of the respective OAR. Furthermore, *n* is the volume exponent and determines whether the organ is of parallel (*n* = 1) or serial type (*n* = 0). If *n* equals 1, the *EUD* is simply given by the mean dose of the organ at risk. *D*_50%_>, *n*, and *m* are empirical fit parameters and can be found in literature for the relevant OAR (46, 47). In **Table 2**, these parameters are listed for the endpoints symptomatic and radiation pneumonitis of the lung. The *NTCP* curves are plotted in **Figure 4B**. A sigmoidal shape of the *NTCP* curve is seen—as expected for a typical dose–response relationship. We use Matlab and the free software extension CERR (48) to determine the respective *NTCP* of each patient depending on the summation dose distributions and DVHs given by Pinnacle and MIM.

#### 2.6.4 Linear Approach by Darby et al. for *NTCP* Calculation of the Heart

Darby et al. empirically investigated the risk of ischemic heart disease of women after breast cancer radiotherapy (42). They conducted a population-based case–control study of major coronary events (MCE) and report on a correlation between the excess relative risk (*ERR*) for the endpoint MCE and the average dose *D*_mean_:


[10] $$ERR=D_{\text{mean}}\cdot 0.074{\text{ Gy}}^{-1},$$

where


[11] cumulative risk=baseline risk ·(1+ERR).

The rate for cardiovascular events increases linearly by +7.4% per Gy in dependency on the mean dose without threshold. The increase of *ERR* begins within a few years after exposure and continues for at least 20 years.

### 2.7 Data Analysis

Statistical analysis was performed using OriginPro 2019b (V. 9.6.5.169, OriginLab Corporation, Northampton, MA, USA). In all presented boxplots, the coloured area depicts the range from 25% to 75% of the data. The error bars correspond to the 1.5 interquartile range. The line dividing the box into an upper and lower part represents the median value. A black circle marks the mean value. All statistical outliers are shown as crosses. We first check if the given data are normally distributed. If true for both samples, we use a Student’s t-test including a Welch correction to assess for statistically significant differences between the cohorts. For non-gaussian data, Wilcoxon’s test was applied. Statistical significance was presumed for p < 0.05. In **Tables 2**–**5** the mean values and their respective standard deviation are shown. Furthermore, the range of values (min–max) is given inside the brackets.

**Table 2:** *EAR* and *NTCP* calculation parameters.

model	organ at risk					
Schneider (32) *LNT and full model*		*δ * [(10,000 PY Gy)^-1^]	*γ*_e_	*γ*_a_	*α* [Gy^-1^]	*R*
	*lung*	8.0	0.002	4.23	0.042	0.83
	*breast*	8.2	-0.037	1.70	0.044	0.15
**LKB**		**endpoint**		***n***	***m***	***D***_**50%**_[Gy]
	*lung*	symptomatic pneumonitis (46)		1.000	0.35	37.6
		radiation pneumonitis (grade ≥ 2) (47)		0.990	0.37	30.8

**Table 3:** Dose exposure for the complete radiotherapy regime as shown in **Figure 6**.

		*scenario 1*	*scenario 2*	*scenario 3*	*scenario 4*
organ at risk	breast size	IMRT + brachytherapy	IMRT + SiB	3D-CRT + brachytherapy	3D-CRT + sequential boost
**heart**	small	1.1 ± 0.3 (0.9–1.6)	1.7 ± 0.4 (1.2–2.2)	0.6 ± 0.2 (0.6–0.9)	1.9 ± 0.7 (0.7–2.6)
	large	3.1 ± 2.8 (1.6–7.8)	1.2 ± 0.4 (0.8–1.9)	1.4 ± 0.5 (1.0–2.1)	1.3 ± 0.5 (0.9–2.0)
**left lung** **(ipsilateral)**	small	3.3 ± 2.4 (1.0–7.1)	2.3 ± 1.4 (0.7–3.6)	1.0 ± 0.7 (0.4–2.1)	1.5 ± 1.5 (0.4–4.0)
	large	18.8 ± 13.9 (7.8–38.3)	10.4 ± 18.3 (1.1–43.0)	12.8 ± 17.2 (1.0–41.5)	12.7 ± 17.1 (1.0–41.2)
**right lung** **(contralateral)**	small	0.4 ± 0.1 (0.3–0.5)	0.5 ± 0.2 (0.3–0.8)	0.2 ± 0.1 (0.2–0.3)	0.5 ± 0.4 (0.3–1.2)
	large	0.6 ± 0.1 (0.5–0.6)	0.4 ± 0.1 (0.3–0.4)	0.4 ± 0.1 (0.3–0.4)	0.4 ± 0.1 (0.3–0.4)
**right breast** **(contralateral)**	small	1.6 ± 0.6 (1.0–2.3)	1.7 ± 0.8 (0.9–2.7)	1.7 ± 0.6 (1.2–2.7)	2.4 ± 1.1 (1.3–3.8)
	large	1.2 ± 1.0 (0.2–2.8)	1.0 ± 0.7 (0.2–2.0)	1.0 ± 0.7 (0.1–2.0)	1.2 ± 1.1 (0.2–3.0)

All TLD measurement values are given for the small and large breasts [in (Gy)].

**Table 4:** Secondary cancer risk for different organs at risk calculated using the TPS data and the TLD measurements.

			*scenario 1*	*scenario 2*	*scenario 3*	*scenario 4*
organ at risk	*age*_x_ [years]	*age*_a_ [years]	IMRT + brachytherapy	IMRT + SiB	3D-CRT + brachytherapy	3D-CRT + sequential boost
**left lung (ipsilateral)** ***EAR*** [10,000 PY^-1^] full model TPS data	55	75	54 ± 13 (42 – 85)	56 ± 8 (45 – 70)	42 ± 7 (36 – 53)	44 ± 8 (30 – 62)
			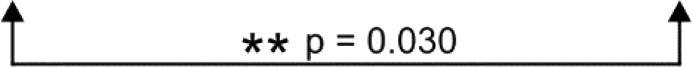			
			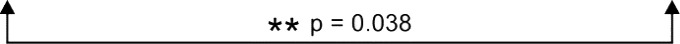			
				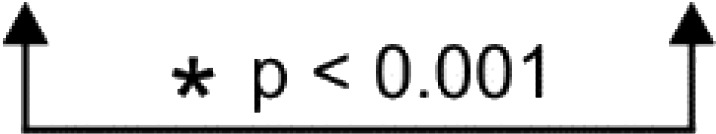		
				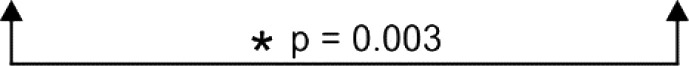		
	55	95	145 ± 35 (114 – 232)	153 ± 21 (122 – 189)	114 ± 18 (96 – 143)	121 ± 22 (81 – 169)
			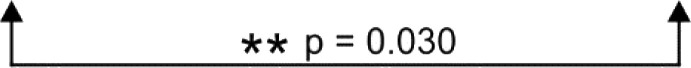			
			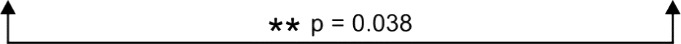			
				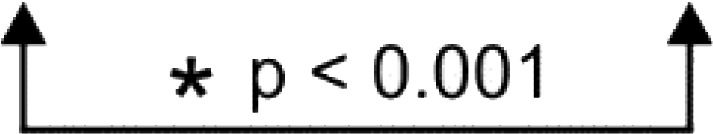		
				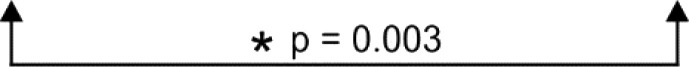		

**right lung (contralateral)** ***EAR*** [10,000 PY^-1^] linear model TLD data	55	75	5 ± 1 (4 – 6)	5 ± 1 (4 – 6)	3 ± 1 (2 – 4)	5 ± 1 (4 – 6)
	55	95	14 ± 3 (11 – 17)	13 ± 2 (11 – 15)	9 ± 3 (6 – 12)	14 ± 3 (11 – 17)
**right breast (contralateral)** ***EAR*** [10,000 PY^-1^] linear model TLD data	55	75	5 ± 1 (4 – 6)	6 ± 2 (4 – 8)	5 ± 1 (4 – 6)	7 ± 2 (5 – 9)
	55	95	8 ± 1 (7 – 9)	9 ± 3 (6 – 12)	7 ± 2 (5 – 9)	10 ± 4 (6 – 14)

*marks a pairwise t-test with p ≤ 0.05 including a Welch correction for different variances.

**marks a pairwise Mann-Whitney U test with p ≤ 0.05.

**Table 5:** Excess relative risk and normal tissue complication probabilities for different organs at risk calculated using the TPS data.

		*scenario 1*	*scenario 2*	*scenario 3*	*scenario 4*
organ at risk	endpoint	IMRT + brachytherapy	IMRT + SiB	3D-CRT + brachytherapy	3D-CRT + sequential boost
**left lung** **(ipsilateral)**	symptomatic pneumonitis	1.9 ± 1.0 (1.1 – 4.3)	2.2 ± 0.7 (1.1 – 3.5)	1.3 ± 0.3 (0.9 – 1.8)	1.5 ± 0.7 (0.7 – 3.3)
***NTCP*** [%] LKB model TPS data			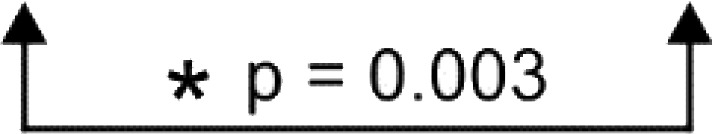		
			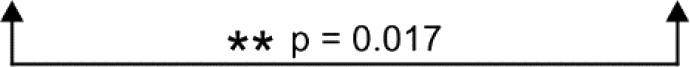		
					
**whole lung** ***NTCP*** [%] LKB model TPS data	radiation pneumonitis (grade ≥ 2)	1.3 ± 0.7 (0.9 – 3.2)	1.5 ± 0.6 (1.0 – 3.0)	1.0 ± 0.2 (0.7 – 1.3)	1.0 ± 0.3 (0.6 – 1.7)
		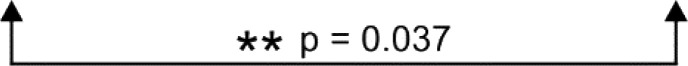			
		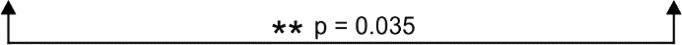			
			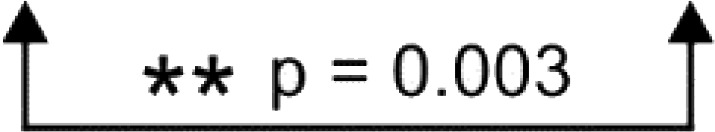		
			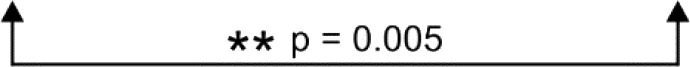		
				
	symptomatic pneumonitis	2.3 ± 1.0 (1.7 – 4.9)	2.6 ± 0.8 (1.8 – 4.6)	1.7 ± 0.3 (1.4 – 2.2)	1.8 ± 0.4 (1.2 – 2.9)
		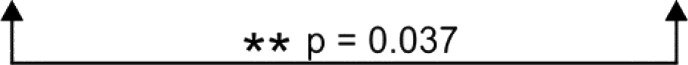			
		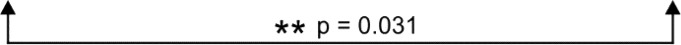			
			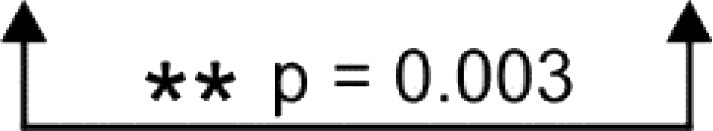		
			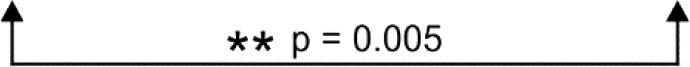		
					
**heart** ***ERR*** [%] Darby model TPS data	ischemic heart disease	+33.4 ± 9.9 (24.3 – 58.7)	+32.8 ± 7.2 (20.6 – 44.8)	+24.5 ± 11.8 (11.4 – 44.6)	+35.2 ± 15.2 (18.1 – 55.9)

*marks a pairwise t-test with p ≤ 0.05 including a Welch correction for different variances. **marks a pairwise Mann-Whitney U test with p ≤ 0.05.

## 3 Results

### 3.1 Dosimetric Comparison of TPS Treatment Plans

**Figure 5:**
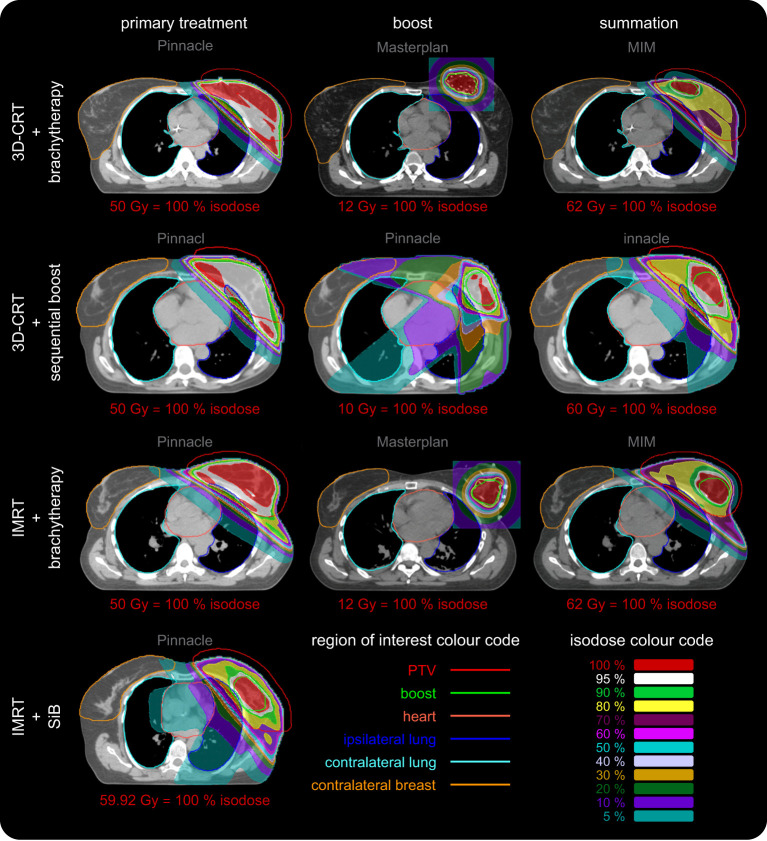
Representative dose distributions of all four regarded scenarios for primary treatment, boost, and their summation. The dose distributions were calculated using Philips Pinnacle and Oncentra Masterplan. The screenshots were made in Pinnacle and MIM. The colour codes of the regions of interest and isodose curves are depicted in the lower right corner. The corresponding dose value of the red 100% isodose is given for each image separately. Each row depicts a different scenario. The columns represent the primary treatment (dose calculation in Pinnacle, left column), the additional boost (dose calculation in Pinnacle and Masterplan, centre column) and their summation (Pinnacle and MIM, right column). The scenario “IMRT + SiB” only has one column since this concept utilises an integrated boost. We use the software predefined windowing “breast” (Pinnacle) and “mediastinal” (MIM) to illustrate the local Hounsfield units or mass density distribution, respectively, as a grayscale. The brachytherapy CT image used to calculate the dose in Masterplan is presented after the deformed registration in MIM. The dose summation is shown without taking into account the BED in order to maintain visual comparability of all scenarios since “3D CRT + sequential boost” and “IMRT + SiB” do not show BED summation as well. In the latter case, direct comparability is not possible since the fractionation schemes are combined into one concept. The various OARs are highlighted as contours: the PTV (red line), the boost volume (green line), the heart (light red line), the left and the right lung (blue and teal line), and the contralateral breast (orange line). The dose distributions are depicted as coloured areas. See inset in the lower right corner for the colour code of the isodoses. The colour scale is normalised to the respective prescribed dose of each case and given below each image. The required minimum target volume coverage (PTV or boost) for clinical acceptance is at least 95 % (white areas), which is fulfilled for all primary WBI and boost treatments. Usually the lowest isodose scale is chosen to be 10 %. We also show the 5 % isodose area to visualise an approximation to the low-dose regime (additional scattering effects are not included) relevant for secondary cancer risk calculation and affected OARs in this respect: the heart, the ipsilateral and contralateral lung and the contralateral breast.

**Figure 6:**
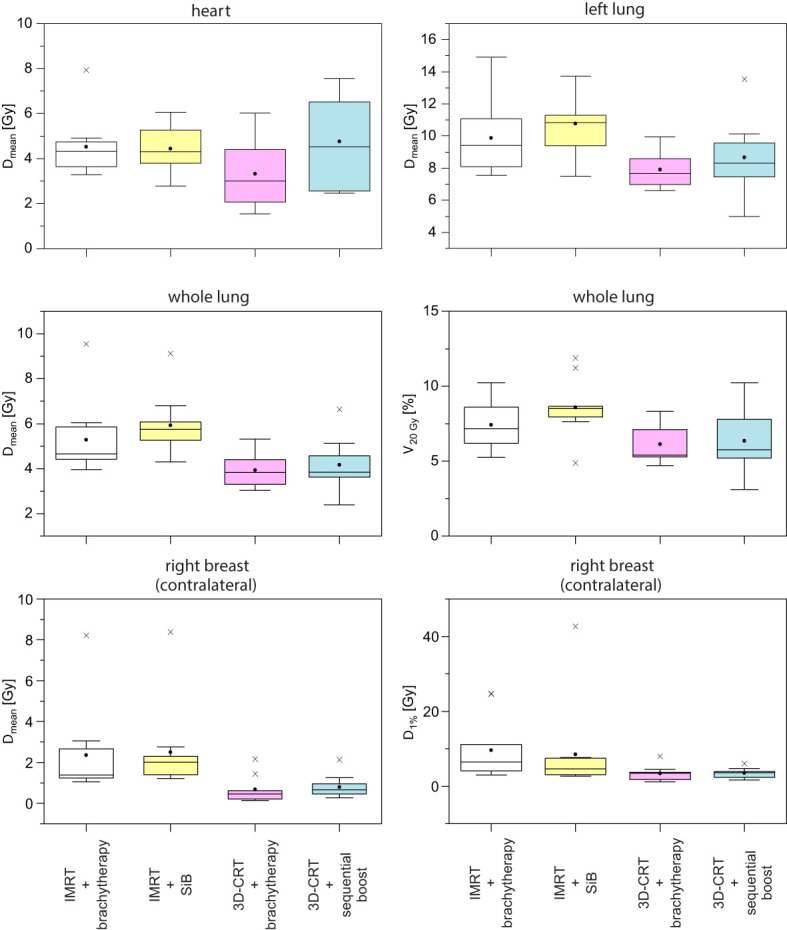
Corresponding boxplots of the data and objectives given in **Table 1**.

Example dose distributions for the four planning scenarios are shown in **Figure 5** and the respective statistical analysis is depicted in **Figure 6**. The objective for the mean dose *D*_mean_ regarding the left and whole lungs is achieved in all cases. Moreover, the objective for the relative volume *V*_20 Gy_ which receives 20 Gy or more, is satisfied as well. The average dose to at least 1% of the contralateral breast ranges between 3.4 and 9.6 Gy. The maximal value in the scenario “3D-CRT + brachytherapy” (42.7 Gy) shows that it was not always possible to place the beam directions so as to completely exclude the contralateral breast for all patients (as is generally desired). However, only a small portion of the contralateral breast (if at all) is penetrated by the primary radiation, and the average mean dose ranges between 0.7 and 2.5 Gy as calculated by the TPS (compare TLD measurements below). However, regarding the ranges of the values, the high-dose outliers (maximum *D*_1%_ of 24.7 Gy for “IMRT + brachytherapy” and 42.7 Gy for “IMRT + SiB”) occur in the IMRT-based scenarios, while the 3D-CRT plans have values < 10 Gy for all patients included in the study (maximum *D*_1%_ 8.0 Gy for “3D-CRT + brachytherapy” and 6.1 Gy for “3D-CRT + sequential boost”). The mean heart dose falls outside the desired range below 3 Gy, while remaining below 5 Gy. In fact, at the time the patients were treated, a 5-Gy mean heart dose in left-sided breast cancer was considered the acceptable limit, while <3 Gy was aimed for if possible. Therefore, the plans were accepted for clinical treatment despite this shortcoming. No statistical significance was found in the pairwise comparisons.

**Figure 7:**
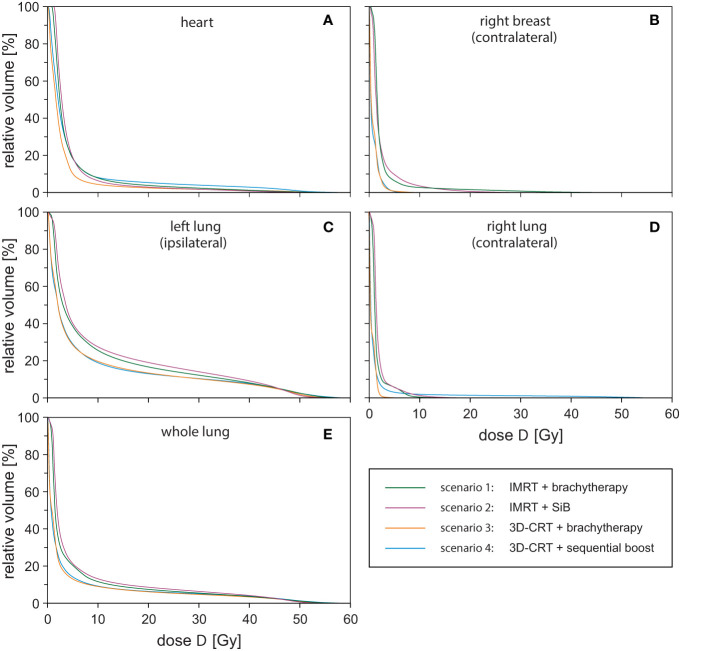
Dose-volume-histograms for all investigated treatment techniques averaged over the respective sub-cohort and all regarded organs at risk: heart **(A)**, contralateral breast **(B)**, ipsilateral lung **(C)**, contralateral lung **(D)**, and whole lung **(E)**.

To summarise the scenarios and estimate the dose exposure on the relevant OARs, we calculated the respective mean DVHs (**Figure 7**). Each DVH shown has been averaged over the respective sub-cohort (see legend of **Figure 7**). In the following, we choose the points *D*_20%_ and *D*_10%_ as well as the regions *D* < 5 Gy and *D* > 10 Gy to characterise the DVH curve shape. Regarding the heart (**Figure 7A**), we find *D*_20%_ > 4.9 Gy and *D*_10%_ > 8.2 Gy for all curves. The highest-dose exposure of the heart in the region *D* < 5 Gy is exhibited by scenario “IMRT + SiB” in contrast to *D* > 10 Gy, where “3D-CRT + sequential boost” is highest. Overall, the heart DVH curve for “3D-CRT + brachytherapy” lies lowest.

For the contralateral breast (**Figure 7B**), we find *D*_20%_ > 2.7 Gy and *D*_10%_ > 4.8 Gy. In general, “IMRT + SiB” shows the highest values; “3D-CRT + brachytherapy” and “3D-CRT + sequential boost” are approximately identical and have the lowest-dose exposure in this range. In **Figure 7C**, the DVH of the ipsilateral lung is depicted. The parameters are *D*_20%_ > 19.0 Gy and *D*_10%_ > 38.4 Gy for all scenarios. Here, the DVH of scenario “IMRT + SiB” lies above all other scenarios. “3D-CRT + brachytherapy” and “3D-CRT + sequential boost” are approximately identical and lowest. The contralateral lung in **Figure 7D** indicates the DVH parameters *D*_20%_ > 2.2 Gy and *D*_10%_ > 3.1 Gy. For *D* < 5 Gy, “IMRT + SiB” and, for *D* > 10 Gy, “3D-CRT + sequential boost” are the ones with the highest exposure. **Figure 7E** illustrates the average DVHs of the whole lung. Here, *D*_20%_ > 5.5 Gy and *D*_10%_ > 15.4 Gy. Furthermore, “IMRT + SiB” shows the highest curve while “3D-CRT + brachytherapy” and “3D-CRT + sequential boost” are almost identical and lowest.

### 3.2 TLD Measurement Results

**Figure 8:**
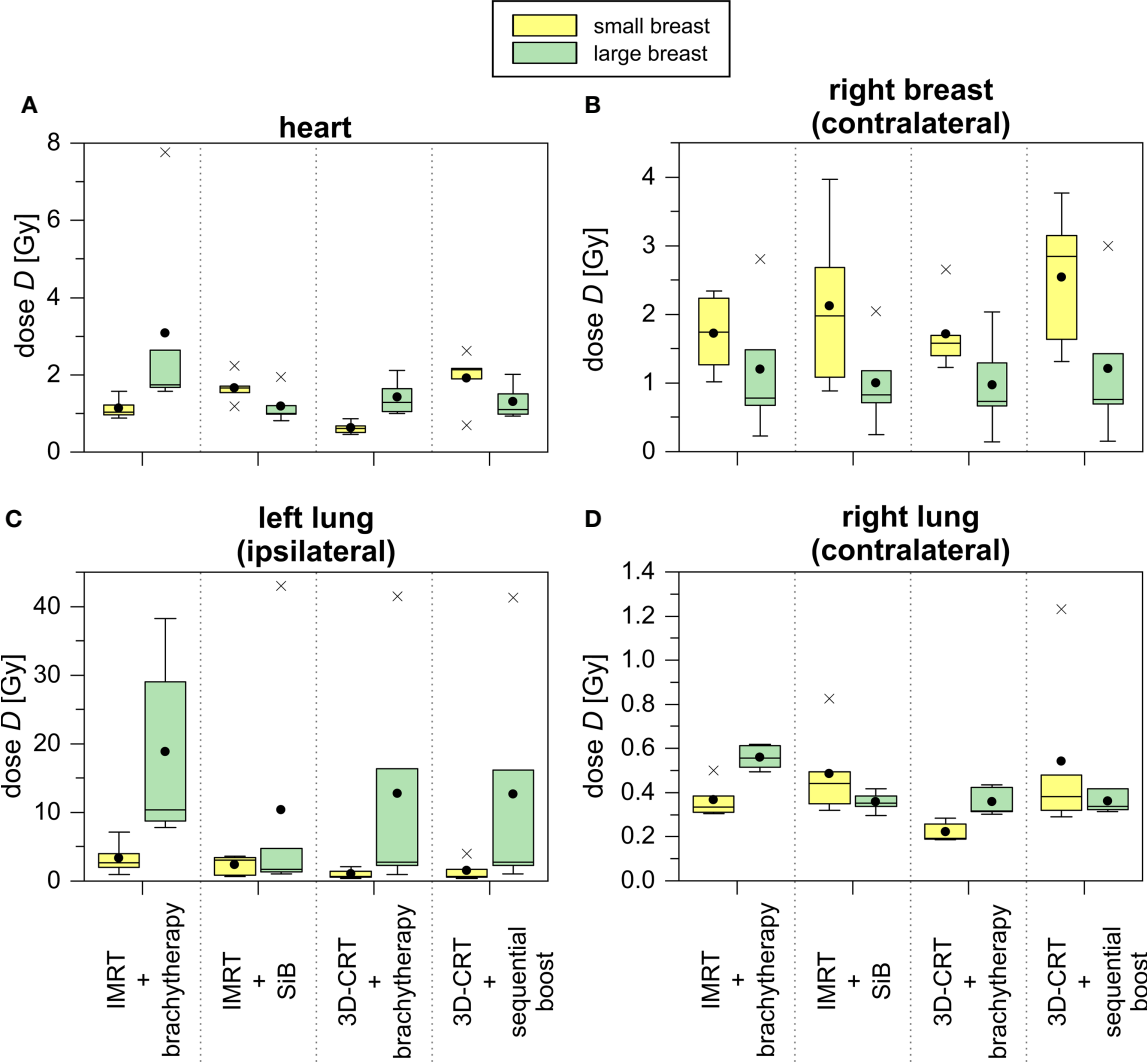
TLD measurements of treatment plans concerning the representative small (yellow) and large (green) breast treatment plans for all scenarios depicted as boxplots. The TLDs were located in the heart **(A)**, the contralateral breast **(B)**, the ipsilateral **(C)** and contralateral **(D)** lung.

The point dose measurements of each OAR were averaged to give the mean organ doses per fraction. Next, the values are multiplied by the number of fractions to obtain the dose exposure of the complete radiotherapy regime (see **Table 3** and **Figure 8**). This allows for comparison to the high-dose in-field values given above. Amongst the four different scenarios, no statistically significant differences could be found. Subsequently, we compared the results for the small and large breast attachments.

The TLD measurements of the heart yield a 2.5 to 5.5 times smaller average dose exposure compared to the in-field TPS calculations. This is also caused by the given field orientation. The fields are touching the heart’s region of interest at its outer edge, and thus, the centrally located TLDs do not measure the in-field part of the primary treatment. Only the scattered out-of-field radiation and the simultaneously integrated as well as the sequential boost are detected (see **Figure 5**). Comparing the achieved values of the small and large breasts, we find a statistical significant difference only in the case “3D-CRT + brachytherapy” (small breast 1.1 ± 0.3 Gy vs. large breast 3.1 ± 2.8 Gy, p = 0.016). For the ipsilateral lung, the TLD measurements of the large breast show a large standard deviation due to an outlier, i.e., one TLD position located just barely in-field. In case the outlier is not considered, we obtain the following results for the large breast: 9.0 ± 1.3 Gy for “IMRT + brachytherapy”, 2.4 ± 2.0 Gy for “IMRT + SiB”, 2.0 ± 1.0 Gy for “3D-CRT + brachytherapy”, and 2.0 ± 0.9 Gy for “3D-CRT + sequential boost”. These values are more consistent with the TPS results. Regarding the small breast, the TLD results are smaller by a factor 3 to 8. This is mainly due to the shape of the tangent required to cover the WBI PTV: for a large breast extending more laterally and dorsally than a smaller breast, the beams are angled more towards the dorsolateral direction, thus including a larger portion of the ipsilateral lung. In general, the brachytherapy scenarios appear more favourable for the smaller breasts. However, the difference between the large and small breast only becomes statistically significant in the scenario “IMRT + brachytherapy (p = 0.040). Comparing the TLD to the TPS data and including the standard deviations, the calculations and measurements match in all cases.

In summary, to model secondary cancer risk adequately only the OARs and their TLDs which are placed completely out-of-field are suitable. Thus, the heart and the ipsilateral lung are considered to be in-field and we use the TPS data for *EAR*, *ERR*, and *NTCP* calculation of these OARs as shown in Section 3.3. Furthermore, the TLD measurements determine the secondary cancer and normal tissue complication risks of the contralateral lung and contralateral breast.

### 3.3 Secondary Cancer Risk and *NTCP*

**Figure 9:**
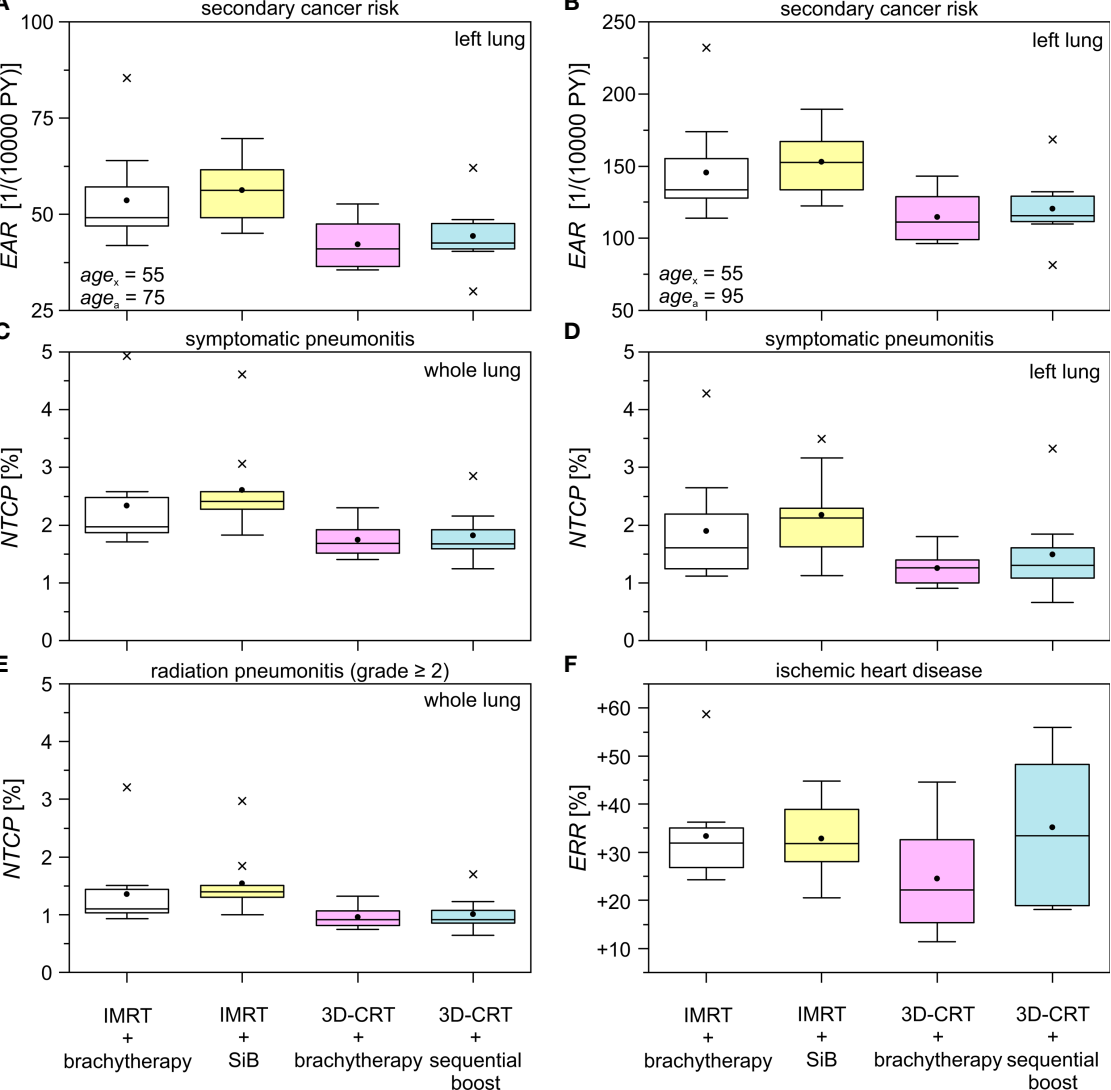
Excess absolute risk *(EAR)*, normal tissue complication probability *(NTCP)*, and excess relative risk *(ERR)* for different treatment techniques and boost concepts as boxplots. The data is shown for the ipsilateral lung in the high dose range using the obtained dose distributions in Pinnacle & MIM. The difference in age between the mean exposed (*age*_x_) and the hypothetically attained age (*age*_a_) is 20 years **(A)** and 40 years **(B)**, respectively. The investigated complications are symptomatic pneumonitis of the whole **(C)** and the left **(D)** lung, radiation pneumonitis (grade ≥ 2) of the whole lung **(E)**, and the endpoint ischemic heart disease **(F)**. See **Tables 4**, **5** for the respective statistical significances.

In the in-field regions, the *EAR* for developing secondary cancer of the left lung are shown in **Figure 9**, calculated at 20 years (**Figure 9A**) and 40 years (**Figure 9B**) after radiation exposure at age 55 years. The results are listed in **Table 4**. In the first case, the magnitude of the *EAR* is around 50 per 10,000 PY. For a larger attained age after irradiation we obtain is scaled up by a factor of 2-3. We find statistically significant differences only for the cases “IMRT + brachytherapy” compared to “3D-CRT + brachytherapy”, as well as “IMRT + SiB” compared to “3D-CRT + sequential boost”. The *EAR* in the out-of-field regions (contralateral lung) calculated using the TLD measurements are of the order of 3–14 per 10,000 PY for all scenarios. The lowest *EAR* is associated with “3D-CRT + brachytherapy”. The highest secondary cancer risk for the right lung is given by “IMRT + brachytherapy” and “3D-CRT + sequential boost” (without statistical significance). Secondary cancer risk for the contralateral breast ranges between 5 and 10 per 10,000 PY for all scenarios, with the lowest values calculated for “3D-CRT + brachytherapy” and highest for “3D-CRT + sequential boost”. However, statistical significance between the scenarios was not reached for any comparison in the out-of-field range.

The normal tissue complication probabilities for the OAR are shown in **Figure 9** and listed in **Table 5**. The calculated *NTCP* for symptomatic radiation pneumonitis of the left lung (**Figure 9D**) is of the order of 1%–2%, with significantly lower probability for the brachytherapy boost scenarios as compared with the “IMRT + SiB” technique. Statistical significance was reached for comparing “IMRT + brachytherapy” with “IMRT + SiB” and “IMRT + SiB” with “3D-CRT + sequential boost”. The complication risk regarding symptomatic pneumonitis of the whole lung (**Figure 9C**) ranges between 1.7% for “3D-CRT + brachytherapy” and 2.6% for “IMRT + SiB”. For the endpoint radiation pneumonitis grade ≥ 2, we observe the same scenario ranking with a range from 1.0% to 1.5% (**Figure 9E**). Regarding the statistical significance, the pairwise Wilcoxon tests yield statistically significant differences for the comparisons “IMRT + brachytherapy” versus “3D-CRT + brachytherapy”, “IMRT + brachytherapy” versus “3D-CRT + sequential boost”, “IMRT + SiB” versus “3D-CRT + brachytherapy”, and “IMRT + SiB” versus “3D-CRT + sequential boost”. In **Figure 9F**, the excess relative risk for a heart disease is depicted (also see **Table 5**). Regarding major coronary events, the average *ERR* ranges from approximately +25% to +35% for the different planning scenarios. No significances could be observed for the *ERR* comparisons.

### 3.4 IMRT Versus 3D-CRT Treatment Methods

To assess the contribution of the percutaneous radiotherapy technique, we now compare the 3D-CRT and the IMRT primary treatment methods in the in-field region independently of the boost technique. However, it must be kept in mind that averages are calculated by combining the “IMRT + brachytherapy” and “IMRT + SiB” scenarios on the one hand and combining the “3D-CRT + brachytherapy” and “3D-CRT + sequential boost” scenarios on the other hand, which means that very heterogeneous groupings are artificially created. Therefore, the absolute numbers are hardly representative; the emphasis here should be on the question of statistically significant differences. For calculation of the p-value, we use the Wilcoxon test.

Considering the secondary cancer risk for the left lung in the in-field region, IMRT shows a significantly higher *EAR* than the 3D-CRT treatment (p < 0.001), namely, 55 ± 10 versus 43 ± 7 per 10,000 PY for 20 years difference in age and 149 ± 28 versus 118 ± 20 per 10,000 PY for an age difference of 40 years. In the out-of-field region, there are no statistically significant differences between the IMRT and 3D-CRT scenarios. Nevertheless, regarding only the mean values, 3D-CRT tends to have a lower *EAR* and *ERR* compared with IMRT.

The normal tissue complication risk of a symptomatic pneumonitis of the left lung is significantly lower for 3D-CRT (1.4 ± 0.6%) compared to IMRT (2.0 ± 0.8%) with p = 0.003. For the total lung and the endpoint symptomatic pneumonitis, we obtain the following results: IMRT 2.5 ± 0.9% versus 3D-CRT 1.8 ± 0.4% (p < 0.001). The risk of a radiation pneumonitis (grade ≥ 2) is 1.4 ± 0.6% for IMRT and 1.0 ± 0.2% for 3D-CRT. These results are statistically significant (p < 0.001). The excessive relative risk of an ischemic heart disease is +33.1 ± 8.4% (IMRT) and +30.4 ± 14.5% (3D-CRT) without a significant difference.

### 3.5 Brachytherapy Versus Teletherapy Boost Concepts

In parallel to the above section, we compare the teletherapy boost concepts against dose saturation of the tumour bed using brachytherapy. Again, we place our main focus on the statistical comparison rather than the aggregated mean values.

The *EAR* calculation of the left lung results in a non-significant difference between both concepts: 48 ± 12 per 10,000 PY (brachytherapy) and 50 ± 10 per 10,000 PY (teletherapy) for *age*_a_ = 75 years (for an age difference of 40 years, 131 ± 32 per 10,000 PY for brachytherapy and 137 ± 27 per 10,000 PY for teletherapy). Regarding the out-of-field TLD measurements and *EAR* calculations for contralateral lung and breast cancer, again no statistical significance is found.

Next, the *NTCP* data are considered. The risk of symptomatic pneumonitis of the left lung is 1.6% ± 0.8% for brachytherapy boosts and 1.8% ± 0.8% for teletherapy boosts (no statistical significance). Symptomatic pneumonitis for the total lung is determined to be 2.1% ± 0.8% for brachytherapy boosts and 2.2% ± 0.7% for teletherapy boosts (no significant difference). Moreover, a radiation pneumonitis has a risk to appear in 1.2% ± 0.5% (brachytherapy) and 1.3% ± 0.5% (teletherapy) of all cases. Also, the risk for developing ischemic heart disease is not significantly different between the scenarios.

Irrespective of the non-given significances, a tendency regarding the average doses, normal tissue complication probabilities, and secondary cancer risks is recognisable (see **Figures 6**, **9**): the scenarios including a brachytherapy boost show smaller results/risks than the scenarios with the same primary WBI treatment and a teletherapy boost (“IMRT + brachytherapy” versus “IMRT + SiB” and “3D-CRT + brachytherapy” versus “3D-CRT + sequential boost”).

## 4 Discussion

In this study, we have presented dose distributions of biologically accumulated BED2 doses for the complete adjuvant radiotherapy regime for patients with left-sided breast cancer, including WBI by either percutaneous tangential 3D-CRT or IMRT and a boost applied either sequentially by 3D-CRT, simultaneously with the IMRT, or as multicatheter interstitial brachytherapy afterloading technique. For the out-of-field region, TLD measurements were made inside an anthropomorphic phantom with breast attachment for two representative patient plans out of each group—one with a large (1,200 cc) and one with a small (350 cc) breast. To take into account the considerable breast deformation which occurs on catheter insertion, 3D-printed phantoms were designed for the application of the brachytherapy dose distributions. To our knowledge, this is the first study to present such a detailed comparison of doses for the different scenarios, including the resulting *NTCP*, *ERR*, and *EAR* modelled by the most widespread approaches. In particular, only few studies have yet included boost doses in the comparison, and to our knowledge, SiB vs. brachytherapy boost scenarios have not been contrasted so far.

### 4.1 Strengths and Limitations of this Study

This study has several limitations: first of all, the relatively small number of 38 patients and the smaller sub-selection of plans for TLD measurements. Only one measurement per breast size and per scenario was performed; however, the patient anatomies for these plans were selected for optimum agreement amongst the patients and with the phantom breast attachments. An increased number in patients and measurements would increase the significance of this study. Moreover, the comparability between the patients and scenarios is not absolute, since we do not calculate and measure four treatment plans (one per scenario) for each patient but rather compare different cohorts. We explicitly decided against performing a planning study and decided to retrospectively review real patient treatment plans from a collective of 38 patients. For all these patients, the same technical equipment was available (planning CT, linear accelerators, afterloading brachytherapy unit, treatment planning systems). Admittedly, this approach introduces some heterogeneity between the cohorts and precludes a pairwise comparison with linked samples; plausibly, statistical significance will be more difficult to achieve since the variance of the dose distributions is larger. A planning study would allow to assess what can optimally be achieved by the different technical approaches in a uniform hypothetical setting and hence identify the most beneficial treatment scenario for each patient—however, this would in any case not be possible before the individual clinical treatment decision, since a planning CT with multicatheter implant would be required for such a comparison. In contrast, the advantage of our approach is that it reflects the clinical reality; these are not treatment plans optimised for study purposed in direct comparison, rather, they represent the standard that was actually accepted by the radiation oncologists for each technique and thereby the true treatment of our patients over that time. The collectives are adequately matched without any significant difference in breast and PTV volume amongst the cohorts.

Some OARs extend from the in-field or penumbra region to the low-dose region beyond the field edge. For these, both the TLD measurements and the TPS calculations present only part of the truth. However, as has been shown for these cases, the TPS and TLD results agree relatively well in these special cases. In clinical practice, the combination of these risk estimates will come closer to the truth than either one separately: the *NTCP* will be mostly influenced by the higher isodoses and can therefore realistically be approximated by the TPS calculations. The secondary cancer risk may be higher with higher isodoses in those parts of the lungs and heart exposed to in-field or penumbra doses, and this is where Schneider’s model should be applied. However, the larger parts of these organs will receive small out-of-field doses, and in these regions, the linear model using TLD measurements should realistically reflect the secondary cancer risk. A more relevant limitation in the risk models arises from the fact that other patient-specific parameters are not included in the models, such as the influence of chemotherapy such as anthracyclines on cardiac toxicity or smoking status on lung cancer. The modelled values therefore reflect only the additional risk posed by radiotherapy, assuming all other factors to remain equal throughout the patient collectives.

A final issue is the neglect of neutrons, which emerge in 18-MV photon irradiation. To make a rough estimate of the neutron contribution, we assessed which percentage of the total prescribed dose was applied by 18-MV beams. Depending on the patient and scenario, this was between 0% and 17%. Using a conservative maximum of 20% of the plan dose delivered by 18-MV photons, this amounts to around 12 Gy. Vanhavere et al. (49) estimate the organ-equivalent doses in prostate treatment, finding a secondary neutron exposure for directly adjacent OAR (the bladder in their case) of approximately 8 mSv per 2 Gy (= 4 mSv/Gy) for 3D-CRT and a Varian Clinac 2100 C-D linear accelerator. In our case, this would result in 48 mSv for 3D-CRT. Secondary neutron generation also depends strongly on the treatment technique. IMRT usually requires more MUs compared with 3D-CRT, and thus, the fluence for IMRT is estimated approximately three times larger than for 3D-CRT (50). With the values above, we obtain 144 mSv for the IMRT treatments. In reality, this is a drastic overestimation since only a minority of plans in our collective used 18 MV for IMRT treatment at all. In principle, it is our institutional policy to avoid IMRT with photon energies higher than 6 MV, and exceptions are only rarely made when the patient’s anatomy is very difficult and adequate plans cannot be achieved with 6-MV photons only. Still, we will carry out the conservative estimate, assuming a 144-mSv neutron dose for IMRT plans and 48 mSv for 3D-CRT. Neutron generation is also dependent on the linear accelerator used, e.g., a Varian 21EX machine with an 18-MV nominal photon beam energy emits approximately 2.5 times more neutrons than a Siemens ONCOR, which shows a relatively flat photoneutron spectrum (51). Including this, we end up with a maximum of 58-mSv total neutron dose for the worst-case scenario IMRT plans. Comparing this value to the prescribed dose of 60 Gy results in approximately 1‰. Thus, secondary neutrons were neglected in our case. This approximation is in line with the conclusions by (49), who stated that secondary neutron influence on *NTCP* and secondary cancer risk are small when compared with gamma radiation.

### 4.2 Comparison with Previous Studies

Comparing the different plan scenarios, we find for the primary course of radiotherapy treatment without boost a reduced *EAR* and *NTCP* for 3D-CRT as opposed to IMRT. The reason is that the incident angles of the tangential fields are chosen in such a way that the left lung, the heart, and the contralateral breast are maximally shielded. In comparison, the IMRT—even with a fan-shaped beam arrangement—uses more widespread angles to obtain a sufficient number of free parameters for the inverse optimisation. While optimising PTV coverage, this leads to an increase in organ and normal tissue risks in those areas which were formerly completely blocked out.

#### 4.2.1 Dosimetric Comparison

A number of studies have presented a dosimetric comparison for tangential 3D-CRT and IMRT planning techniques, considering the primary course of treatment without additional boost: in **Table 6**, several recent literature dose values are summarised for free-breathing techniques and 50-Gy whole-breast irradiation. While not always achieving our aim of reducing the mean heart dose to below 3 Gy, our dose values for the summation plans remain within the range observed by other authors. In particular, it has been observed that the mean heart dose reported has steadily decreased over the past years (52, 53), which may reflect greater emphasis on the heart sparing in the light of the elevated risk of ischemic heart disease and better technical possibilities to achieve improved OAR sparing in adjuvant treatment of left-sided breast cancer, such as deep-inspiration breath hold (DIBH) (54, 55) or respiratory-gated treatment (56, 57), prone vs. supine patient immobilisation (58), VMAT (59, 60), or even proton treatment (61).

**Table 6:** Recent findings in literature compared to our results.

study	heart*D*_mean_ [Gy]	whole lung*D*_mean_ [Gy]	whole lung*V*_20 Gy_ [%]	ipsilateral lung*D*_mean_ [Gy]	comment
**Xie 2020** (56)	*3D-CRT* 9.6 ± 3.7 *IMRT* 7.4 ± 1.3 (n.s.)	*3D-CRT* 6.7 ± 1.0 *IMRT* 5.9 ± 0.9 (p < 0.05)	*3D-CRT* 12.7 ± 2.0 *IMRT* 7.9 ± 2.6 (p < 0.05)		
**Supakalin 2018** (58)				*3D-CRT* 9.5 ± 1.8 *IMRT* 7.7 ± 1.4 (p < 0.001)	Left- and right-sided breast cancer
**Salvestrini 2022** (49)	1.3 - 7.2 without information on technique				Review of mean heart doses in free breathing
**Vikström 2018** (54)	*3D-CRT* 6.2 ± 4.4			6.8 ± 1.2	
**Saini 2019** (52)	1.88			6.1	
**Sripathi 2017** (59)	*3D-CRT* 7.1 ± 3.0 *IMRT* 11.94 ± 1.73	*3D-CRT* 16.4 ± 4.4 *IMRT* 20.2 ± 1.3	*3D-CRT* 31.4 ± 10.8 *IMRT* 35.3 ± 7.5		
**Johansen 2011** (77)	6.5 ± 4.7			13.7 ± 1.9	*3D-CRT*
**Edvardsson 2015** (50)	2.5	5.4	9.1		*3D-CRT*
**Tommasino 2017** (55)	3.5			7.6	*IMRT*
**Kuo 2021** (51)	3.42 ± 1.20			8.81 ± 1.33	Tangential *IMRT*, medium expiration
**Corradini 2018** (62)	2.54 ± 1.40	3.6 ± 0.8			*3D-CRT*
**Haciislamoglu 2019** (65)	*3D-CRT* 4.41 ± 2.2 *IMRT* 8.40 ± 2.54			*3D-CRT* 7.24 ± 2.59 *IMRT* 12.58 ± 1.75	*3D-CRT* was planned as field-in-field
**Stewart 2008** (78)	3.5	6.5	10		*3D-CRT*
**Taylor 2017** (57)	5.2	5.7		9.0	Systematic review (2010–2015)
**This study**	*3D-CRT + boost* 4.8 ± 2.1 *3D-CRT + brachy* 3.3 ± 1.6 *IMRT + SiB* 4.4 ± 1.0 *IMRT + brachy* 4.5 ± 1.3	*3D-CRT + boost* 4.2 ± 1.1 *3D-CRT + brachy* 3.9 ± 0.8 *IMRT + SiB* 5.9 ± 1.3 *IMRT + brachy* 5.3 ± 1.6	*3D-CRT + boost* 6.3 ± 2.0 *3D-CRT + brachy* 6.1 ± 1.3 *IMRT + SiB* 8.6 ± 1.9 *IMRT + brachy* 7.4 ± 1.6	*3D-CRT + boost* 8.7 ± 2.2 *3D-CRT + brachy* 7.9 ± 1.1 *IMRT + SiB* 10.8 ± 1.8 *IMRT + brachy* 9.9 ± 2.3	Total dose including boost/SiB

n.s., not significant.

Regarding dose to the lungs, our results are also consistent with previous studies in terms of retrieved dose values. The determined doses can be compared with the report by Taylor et al. (62). They find typical modern whole-lung doses to be 5.7 Gy and whole-heart doses to be 5.2 Gy for left-sided breast cancer treatment. For the comparison of techniques, Xie et al. (63) observed a significantly reduced whole-lung dose for IMRT vs. 3D-CRT; a similar result was obtained by Supakalin et al. (64) for the ipsilateral lung. This is not reflected in our data: the lung dose metrics in our collective are higher for “IMRT + SiB” vs. “3D-CRT + teletherapy boost” (p = 0.033 for the ipsilateral lung, p = 0.005 for the whole lung) and for “IMRT + brachytherapy” vs. “3D-CRT + brachytherapy” (p = 0.033 for the ipsilateral lung, p = 0.023 for the whole lung), respectively. This is also paralleled by the toxicity and secondary cancer risk comparisons presented in the following. The underlying reason may be that even the tangential fanned beams in the IMRT scenarios and more specifically the more widespread beams in the “IMRT + SiB” plans traverse a larger portion of the contralateral lung and breast than the tangents that were specifically designed to block out these organs at risk in the 3D-CRT plans. However, we have not assessed whether this is counterbalanced by improved PTV and boost coverage or conformity, as has been suggested in a number of studies (60, 63, 65–67). We did not include this comparison owing to the different delineation of teletherapy and brachytherapy targets. Besides, the brachytherapy plans are usually created and accepted for clinical use without considering a biological summation dose including the preceding percutaneous WBI as would be done for percutaneous WBI + boost, so in clinical practice these plans are differently evaluated in terms of quality metrics.

For the contralateral breast, for the most part this is not comprised inside the treatment fields and will mainly receive scattered doses as measured with the TLDs (of the order of 1 Gy for the large and up to 2 Gy for the small breast attachment). However, for some treatment plans, a small medial wedge of the right breast may be traversed by a treatment beam making up the edge of the tangent. Here the calculated doses from the TPS yield a mean contralateral breast dose between 0.7 and 2.5 Gy (higher for IMRT vs. 3D-CRT [p < 0.001] and for teletherapy vs. brachytherapy boost [n.s, not significant]), which corresponds adequately with the TPS predictions, and dose maxima in the right breast of the order of 3.4 Gy (3D-CRT + brachytherapy) to 9.6 Gy (IMRT + brachytherapy). The maxima evidently correspond to the in-field part of the contralateral breast.

Only few studies have investigated out-of-field organ doses for breast radiotherapy using TLD measurements. For the 50-Gy total dose, Williams et al. (68) measured contralateral breast doses in the range of 13–60 cGy for 3D-CRT and 103–124% of this for IMRT, somewhat lower than reported by Vlachopoulou et al. (69) for 3D-CRT (1.0 ± 0.4 Gy). Similarly, Behmadi et al. (70) obtained doses of 17.7–213.2 cGy for the ipsilateral lung, 14.8–31.6 cGy for the contralateral lung, and 53.6–134.2 cGy for the heart (again, for 50-Gy WBI using 3D-CRT). When scaled to the higher total dose, these results are in good agreement with ours, allowing for some deviations by the inclusion of the boost and the combination of different treatment techniques.

#### 4.2.2 *NTCP* Models

As most studies regarding treatment techniques and toxicity after breast cancer radiotherapy have concentrated on the whole-breast treatment series (25 × 2 Gy or 28 × 1.8 Gy), we will first compare *NTCP* and *EAR* results for these scenarios before moving on to the complete treatment series including boost which we considered in this manuscript.

Using a tangential 3D-CRT beam setup for 50 Gy WBI, Edvardsson et al. (57) modelled an excess cardiac mortality probability of 0.49% and a risk of developing radiation pneumonitis of 0.31%. For the same fractionation applied using IMRT, Tommasino et al. (61) estimated a risk for a major coronary event of 2.0%, assuming a baseline cardiac risk without radiotherapy of 1.6%. Calculating our way back from the *EAR* to the *ERR* with this baseline risk, this corresponds to an *ERR* of 25%, which is similar to our estimates. Correspondingly, Corradini et al. (71) observed cardiac *ERR* values of approximately 20% for free-breathing 3D-CRT treatment.

Regarding the risk for symptomatic pneumonitis of the ipsilateral lung, Kuo et al. (56) report an estimate of 10.95% for treatment during mid-lung expansion. The difference between these values and those reported by Edvardsson (0.31%, see ref. 61) is rather large, comprising our range of values (1.3–2.2%).

#### 4.2.3 *EAR* Estimates

The estimates of secondary cancer risk rely on very different models due to the very different dose regimes. In this work, we decided to include the *EAR* for the ipsilateral lung as based on the full model by Schneider et al. (32) applied to the dose distribution from the TPS. The rationale for this choice is that the major contribution to left lung secondary cancer induction can be presumed to arise from the small high-dose tangent through the lung, which is comprised inside the open treatment beams. There will also be large areas of the ipsilateral lung exposed to very-low out-of-field doses, which will not be accurately estimated by the TPS. For this area of the shielded ipsilateral lung, the scattered doses will be considerably lower than the in-field doses and probably still higher than the scattered dose to the contralateral lung, which is completely outside the treatment beams. Therefore, to some degree our estimates represent the two extremes: the calculated TPS-dose-based excess absolute risks *(EARs)* for the left lung based on the mechanistic model on the one hand and the contralateral out-of-field doses measured by TLDs and translated into *EAR* according to the linear model on the other hand. For the ipsilateral lung, the *EAR*s calculated for an age of exposure of 55 years and an attained age of 75 years are between 42 and 56 per 10,000 PY, depending on the treatment plan. Contralaterally, the risk is lower by about an order of magnitude (3–5 per 10,000 PY), which is in concordance with our assumption that the ipsilateral risk will be mainly attributed to the high-dose region.

The influence of different radiotherapy techniques (tangential 3D-CRT, IMRT, VMAT) on secondary cancer risk was investigated by Corradini et al. (71), Karpf et al. (72), and Haciislamoglu et al. (73) for 50-Gy percutaneous WBI. For an age of exposure of 30 years and attained age of 70 years, Haciislamoglu et al. (73) estimate an *EAR* for the contralateral lung of 4.4 ± 0.7, 19.9 ± 3.6, and 19.6 ± 1.9 per 10,000 PY for 3D-CRT, IMRT, and VMAT, respectively. For the ipsilateral lung, they report an *EAR* of 28.3 ± 8.0, 61.7 ± 7.1, and 65.2 ± 5.4 per 10,000 PY and for the contralateral lung of 3.5 ± 0.6, 27.2 ± 4.4, and 21.6 ± 3.3 per 10,000 PY, respectively (3D-CRT, IMRT, VMAT). For all these scenarios, they find a significant advantage for the 3D-CRT plans in comparison with IMRT and VMAT, but no significant difference between the IMRT and VMAT plans. Similarly, Karpf et al. (72) considered an IMRT scenario and used the actual age of exposure of the patients and a hypothetical attained age of 70 years, finding *EAR* values of 27.07 ± 2.18 per 10,000 PY for the ipsilateral lung, 7.13 ± 1.11 per 10,000 PY for the contralateral lung, and 2.99 ± 2.15 per 10,000 PY for the contralateral breast, which agrees comparatively well with our results when allowing for the different fractionation and age at exposure. Corradini et al. (71), comparing 3D-CRT and VMAT for 50 Gy WBI, an age of exposure of 50 years, and an attained age of 70 years, distinguished high- and medium-baseline-risk patients, resulting in a larger variation of modelled *EAR* values for the lung (8–67 per 10,000 PY for 3D-CRT vs. 9–78 per 10,000 PY for VMAT). Allowing for the different assumed ages and the limitation to only WBI, these values are in agreement with our model results. Zhang et al. (65) observe considerably larger *EAR* values than the other studies, which cannot be explained merely by the different assumed ages; however, the relative comparison of treatment techniques (tangential 3D-CRT, tangential two-field IMRT, six-field IMRT, and VMAT) confirms our results and those of the other authors cited, in that tangential 3D-CRT entails lower *EAR* than six-field IMRT, both remaining below the *EAR* of VMAT.

#### 4.2.4 Comparison of the Boost Concepts

Moving on to include the boost concepts, we expect that the normal tissue and OAR are less exposed to ionising radiation due to the given placement of the interstitial catheters inside the target volume and the rapid dose fall-off of the Iridium isotope dose in tissue. The comparison of all four scenarios in Section 3.3 yields a favourable combination: the 3D-CRT primary treatment method and an additional dose escalation using a brachytherapy boost reduces the *EAR*, *ERR* and *NTCP* the most.

To our knowledge, boost concepts have only been considered by a small number of authors so far. A 3D-CRT vs. IMRT planning study for WBI of 50.4 and 1.8 Gy with a sequential boost of 16 Gy was presented by Simonetto et al. (22). These authors report mean organ doses of 1–5 Gy for the contralateral breast, 8–10 Gy for the ipsilateral lung, and 0.3–1 Gy for the contralateral lung, well in line with our results. Hayden et al. (74) and Aly et al. (75), respectively, consider SiB scenarios with different fractionation regimes (50/60 Gy in 25 fractions and 50.5/64.4 Gy in 28 fractions). The former, using an IMRT technique, observe a mean dose to the left lung of 13.41 Gy (compared with 10.8 Gy in our study), a mean heart dose of 6.88 Gy (vs. 4.4 Gy in this work), and a mean contralateral breast dose of 0.63 Gy (calculated in their TPS, compared to 1.56 Gy from our TLD measurements). Aly et al. (75) report a mean ipsilateral lung dose of 8.4 ± 1.6 Gy for 3D-CRT and 9.1 ± 1.5 Gy for a combined IMRT-VMAT approach (p < 0.05), a mean heart dose of 3.0 ± 0.9 Gy (3D-CRT) vs. 3.5 ± 1.0 Gy (IMRT-VMAT, not significant), and a mean contralateral breast dose of 1.1 ± 0.3 Gy vs. 1.2 ± 0.3 Gy (significant). Our results fall well between these two studies. A comparison between a sequential boost (3D-CRT, 50 Gy + 16 Gy in fractions of 2 Gy) and SiB (50/60 Gy in 25 fractions, static fields, or TomoTherapy technique) by Van Parijs et al. (76) reported no significant advantage of either technique for the mean heart dose (3.04, 3.12, and 2.97 Gy, respectively) and ipsilateral mean lung dose (6.26, 6.72, and 6.13 Gy, respectively) but a significant difference in calculated contralateral breast dose (0.36, 0.44, and 1.17 Gy, respectively). These doses correspond to the values we observe in the “3D-CRT + sequential boost” and “IMRT + SiB” scenarios.

Interstitial brachytherapy concepts were presented in 32- to 34-Gy accelerated partial breast irradiation concepts by Novotná et al. (77) and Chatzikonstantinou et al. (78), showing good cardiac dose sparing. As a boost after external beam radiotherapy, only Fröhlich et al. (79) combined interstitial multicatheter brachytherapy (3 × 4.75 Gy) with tangential beam WBI (15 × 2.67-Gy accelerated fractionation), showing that brachytherapy achieved a higher dose to the target with equal sparing of OARs in comparison to percutaneous treatment of both series, which is consistent with our observations, albeit for a normally fractionated dose concept.

#### 4.2.5 Comparison With Clinical Observations

Finally, how do our secondary cancer risk estimates compare with clinical observations? Second solid cancers observed in the SEER cancer registry for >5 year survivors presented by Berrington et al. (80) gave an *EAR* for contralateral breast cancer of 5 per 10,000 PY (and an increased incidence of lung cancer of 0.4% vs. 0.3% for non-irradiated patients, which translates into an *EAR* of 10 per 10,000 PY—compared to Xie et al. (81)). Our model predictions give a plausible approximation of these observational study results, although a precise comparison is difficult due to different evaluated time lags and baseline risks—compared to Pignol et al. (82).

## 5 Conclusion

In this work, we compare four different adjuvant treatment techniques of breast cancer in context of breast-conserving therapy regarding normal tissue complication probabilities and secondary cancer risks: “IMRT + brachytherapy”, “IMRT + SiB”, “3D-CRT + brachytherapy”, and “3D-CRT + sequential boost”. In general, 3D-CRT shows the best risk reduction in contrast to IMRT. Concerning the boost concepts, brachytherapy is the most effective method in order to minimise *EAR* and *NTCP* compared to teletherapy boost concepts. Hence, the 3D-CRT technique in combination with an interstitial multicatheter brachytherapy boost shows the lowest secondary cancer risks and normal tissue complication probabilities for treating breast cancer with techniques including boost concepts. However, these results reflect only the normal tissue effects and do not compare other important endpoints such as the PTV coverage or the tumour control probability.

## Data Availability Statement

The raw data supporting the conclusions of this article will be made available by the authors, without undue reservation.

## Author Contributions

PM and CR were responsible for patient treatment. YD and FN devised and planned the project. PM advised regarding the medical aspects of the project. MV and YD wrote the manuscript based on a first draft of the medical doctoral thesis of JG, which is based on this project. YD and MV supervised the doctoral work by JG, in which JG performed the patient retrieval and review in the database, import into the planning systems, image registration, dose calculations, and export. The dosimetric analysis was facilitated by code provided by MV. The secondary cancer risk model was carried out in a Matlab script by MV. MV created the virtual 3D model and implemented the breast add-on into the setup of phantom and afterloader. BT manufactured both the brachytherapy breast phantoms using his private 3D printer. HA and MS performed the TLD preparation and read-out. JG and MV performed the statistical data analysis. Figures were prepared by MV and JG. All authors read and approved the final manuscript.

## Conflict of Interest

Author BT was employed by Siemens Healthcare GmbH.

The remaining authors declare that the research was conducted in the absence of any commercial or financial relationships that could be construed as a potential conflict of interest.

## Publisher’s Note

All claims expressed in this article are solely those of the authors and do not necessarily represent those of their affiliated organisations, or those of the publisher, the editors and the reviewers. Any product that may be evaluated in this article, or claim that may be made by its manufacturer, is not guaranteed or endorsed by the publisher.
